# Individual and mutual effects of elevated carbon dioxide and temperature on salt and cadmium uptake and translocation by rice seedlings

**DOI:** 10.3389/fpls.2023.1161334

**Published:** 2023-04-05

**Authors:** Yu-Xi Feng, Peng Tian, Cheng-Zhi Li, Qing Zhang, Stefan Trapp, Xiao-Zhang Yu

**Affiliations:** ^1^ College of Environmental Science & Engineering, Guilin University of Technology, Guilin, China; ^2^ Department of Environmental and Resource Engineering, Technical University of Denmark, Kongens Lyngby, Denmark

**Keywords:** *Oryza sativa*, CO_2_, climate change, salinization, phytotoxicity

## Abstract

Plant kingdoms are facing increasingly harsh environmental challenges marked by the coexposure of salinity and pollution in the pedosphere and elevated CO_2_ and temperature in the atmosphere due to the rapid acceleration of industrialization and global climate change. In this study, we deployed a hydroponics-based experiment to explore the individual and mutual effects of different temperatures (low temperature, T1: 23°C; high temperature, T2: 27°C) and CO_2_ concentrations (ambient CO_2_: 360 ppm; medium CO_2_: 450 ppm; high CO_2_: 700 ppm) on the uptake and translocation of sodium chloride (NaCl, 0.0, 0.2, 0.6, and 1.1 g Na/L) and cadmium nitrate (Cd(NO_3_)_2_·4H_2_O, 0.0, 0.2, 1.8, and 5.4 mg Cd/L) by rice seedlings. The results indicated that Cd and Na exposure significantly (*P*< 0.05) inhibited plant growth, but T2 and medium/high CO_2_ alleviated the effects of Cd and Na on plant growth. Neither significant synergistic nor antagonistic effects of Cd and Na were observed, particularly not at T1 or high CO_2_. At increasing temperatures, relative growth rates increased despite higher concentrations of Cd and Na in both rice roots and shoots. Similarly, higher CO_2_ stimulated the growth rate but resulted in significantly lower concentrations of Na, while the Cd concentration was highest at medium CO_2_. Coexposure experiments suggested that the concentration of Cd in roots slightly declined with additional Na and more at T2. Overall, our preliminary study suggested that global climate change may alter the distribution of mineral and toxic elements in rice plants as well as the tolerance of the plants.

## 1 Introduction

Elevated atmospheric CO_2_ concentrations are the driver of global climate change. The CO_2_ “fertilization effect” was observed by enhancing vegetation productivity (Sperry et al., 2019; Song et al., 2022). In fact, elevated CO_2_ stimulates the growth of C_3_ plants in terms of plant biomass, water and nutrient use efficiency, and the rate and intensity of photosynthesis (Poorter et al., 2022). Ample evidence has shown that the optimal atmospheric CO_2_ concentrations for crop photosynthesis range from 700 to 1000 ppm, while a deficiency in the CO_2_ supply under natural conditions is a limiting nutritional factor for crops to produce enough C-containing biomass. However, more emissions of CO_2_ from various sources might cause unexpected consequences, wherein warmer temperatures and changing precipitation patterns caused by greenhouse effects are major concerns, which will change the distribution and availability of freshwater, essentially alter tillage patterns and reduce crop yields in some regions (IPCC, 2019).

Another concern is salinization of soils, particularly in combination with irrigation in agricultural land (Corwin, 2021), since the vapour pressure of water increases exponentially with increasing temperature (Clausius-Clapeyron Law), leading to a higher water vapour deficit and more evaporation from soil surfaces. Therefore, the area at risk of salinization is projected to increase in the future (IPCC, 2019). It has been reported that 16% of agricultural land (831 million hectares) is affected by salinization worldwide (FAO, 2011; Thiam et al., 2021), wherein 99.13 million hectares of land are saline in China (Liu and Wang, 2021). It is known that above a certain plant-specific threshold, soil salinity leads to toxic effects and to declining yields (Syed et al., 2021; Yan et al., 2021). Terrestrial plants maintain their internal osmotic pressure at homeostatic levels (Zhao et al., 2021) and regulate their cytosolic K^+^ to Na^+^ ratio by molecular cation transporters (Cosco et al., 2019). Overloading of these pumps leads to toxic effects (Isayenkov and Maathuis, 2019), while exclusion of Na at the root increases salinization (Wei et al., 2022). Production efficiency (carbon use efficiency) increases at higher growth temperatures (Collalti et al., 2020), but adverse effects of soil salinity intensify (Silva et al., 2013); hence, there is no linear relationship between temperature and salt stress (Gadallah, 1996). Therefore, minimizing water use by regulating the transpiration rate is a positive strategy that can help to conserve water and reduce Na loading in plant materials (Xue et al., 2021). In fact, at elevated CO_2_, plants typically use less water and use it more efficiently, and saving water under salt stress is beneficial (Bertolino et al., 2019). Although many studies have been conducted, the current knowledge about the interaction between elevated CO_2_/temperature and salinity remains insufficient for predicting the impact of climate change on plant growth and production.

Today, heavy metal contamination has become a serious and growing problem for agricultural production and food safety (Fei et al., 2022; Zhang et al., 2022; Feng et al., 2023a). Rice (*Oryza sativa* L.) is one of the most important staple food crops worldwide, especially in eastern Asian countries. Over the last century, farmland deterioration due to metal contamination has become increasingly serious. Many anthropogenic activities, including mineral fertilizers, mining operations, manufacturing industries and landfills of industrial or domestic sludge, have resulted in an increasing amount of agricultural land being contaminated with heavy metals throughout the world (Dai et al., 2019). There are more than 20 million hectares of agricultural land contaminated with various species of heavy metals in China (Peng et al., 2019). Such amounts of heavy metals in soils make their entry into the food chain likely and pose a threat to humans and wildlife. It was estimated that the economic damage due to cadmium (Cd) contamination is at least 798 billion CNY in China alone (Yang et al., 2022). Cadmium is a nonessential element for plants (Abbas et al., 2022) and toxic at low concentrations. In fact, Cd can distribute and damage nutrient uptake, photosynthetic and respiratory activities, and hormone balance to decrease and/or repress plant growth (Rasafi et al., 2020). Although the uptake and translocation of Cd in different species of plants have been intensively studied from laboratory tests to field trials, the interaction between elevated CO_2_/temperature and Cd is currently not available.

The uptake of heavy metals has been observed at elevated CO_2_, mainly focusing on plant growth and development (Guo et al., 2015). For example, elevated CO_2_ was able to enhance the uptake rate of heavy metals by stimulating root development, enriching root phenotype traits, and increasing metal availability in soils (Jia et al., 2018). Additionally, elevated CO_2_ stimulated an increase in the fresh weight of soybean in the presence of Cd exposure by positively regulating flavonoid content and antioxidant defense capacity (Gong et al., 2023). Elevated concentrations of heavy metals and salt (NaCl) often occur together (e.g., in wastewater or in fertilizers, Tornabene et al., 2020), and several studies have addressed their mutual interactions on uptake and toxicity. Several physiological interactions between NaCl and Cd were observed in *Mesembryanthemum crystallinum* L. (Nosek et al., 2020). Combined, salt and Cd had a stronger negative effect on the growth and chlorophyll content of conocarpus (*Conocarpus erectus* L.) than each alone (Rehman et al., 2019). Elevated salinity intensified the effects of Cd in a study with quinoa (*Chenopodium quinoa* Willd.) and reduced the shoot and root growth of experimental plants by more than 50% (Abdal et al., 2021).

Because many biotic and abiotic factors are involved, it is difficult to quantify future trends. Present findings remain inconclusive, and very few studies have identified the effect of multiple stressors on terrestrial vegetation under the combined variation of temperature and CO_2_ concentrations. Until now, the simultaneous effects of changing temperatures and CO_2_ concentrations in air on the uptake, translocation, and toxicity of Na and Cd by rice seedlings have not been available. Therefore, the main objectives of this study were (1) to estimate the effects of temperature and CO_2_ concentrations on the plant growth of rice seedlings under salt (measured as Na) and Cd stress alone and in combination; (2) to investigate the individual and mutual effects of temperature and CO_2_ concentrations on the uptake and translocation of Na and Cd alone and in combination; and (3) to analyse the individual and mutual effects of temperature and CO_2_ concentrations on the metal distribution in rice tissues under Na and Cd stress alone and in combination.

## 2 Methods and materials

### 2.1 Rice cultivars and exposure solutions

This experiment was conducted at Guilin University of Technology on the Yanshan campus, Guangxi, China (25.2895 N, 110.3178 E). The seeds (*Oryza sativa* L. cv. XZX 45, Feng et al., 2023b) of rice were cultivated in sandy soils in a climate-controlled chamber (temperature: 25 ± 0.5°C, relative humidity: 60 ± 2%, ambient CO_2_: 360 ppm). The modified 8692 nutrient solution was prepared in 1.0 L of water with KNO_3_ (285.2 mg/L), MgCl_2_·H_2_O (12 mg/L), CaCl_2_ (18 mg/L), MgSO_4_ (15 mg/L), KH_2_PO_4_ (33.46 mg/L), NaHCO_3_ (150 mg/L), H_3_BO_3_ (0.1855 mg/L), MnCl_2_ (0.415 mg/L), Fe-EDTA (4.83 mg/L), NaMoO_4_ (7 mg/L), CuSO_4_ (6.25 mg/L), ZnSO_4_ (2.99 mg/L), and CoCl_2_ (1.5 mg/L) (Feng et al., 2019). After 16 d of growth, ten young seedlings of similar size were collected and incubated with a modified ISO 8692 nutrient solution for 12 h. After acclimatization, the seedlings were cleaned with ionic removal buffer solution [1 mM CaCl_2_ + 2 mM MES-Tris (pH 6.0)] for 4 h (Ling et al., 2021). Finally, the pretreated seedlings were used for the subsequent experiments. In detail, three different solutions with Cd and Na alone or combined were used as follows:

***Solution A* (*Cd stress*)**: Selected seedlings were exposed to a 50 mL Erlenmeyer flask spiked with 50 mL of Cd at nominal concentrations (0.0, 0.2, 1.8, and 5.4 mg Cd/L);

***Solution B* (*Na stress*)**: Selected seedlings were exposed to a 50 mL Erlenmeyer flask spiked with 50 mL of Na at nominal concentrations (0.0, 0.2, 0.6, and 1.1 g Na/L);

***Solution C* (*Cd+Na stress*)**: Selected seedlings were exposed to a 50 mL Erlenmeyer flask spiked with 50 mL of Cd+Na at nominal concentrations (0.0, 0.2 mg Cd/L+ 0.2 g Na/L, 1.8 mg Cd/L+ 0.6 g Na/L, and 5.4 mg Cd/L+ 1.1 g Na/L).

Note that cadmium nitrate [Cd(NO_3_)_2_·4H_2_O, CAS: 10325-94-7] and sodium chloride (NaCl, CAS: 7647-14-5), guaranteed reagents, were used in this study. A remarkable (*P<* 0.05) increase in IR was observed in rice seedlings from both Cd and Na treatments with different nominal initial concentrations (**Supporting information M1**). Accordingly, the EC was determined by IR (Lin et al., 2020). The EC_20_, EC_50_ and EC_75_ of Cd were 0.14, 1.81 and 5.66 mg Cd/L, respectively, while the EC_20_, EC_50_ and EC_75_ of Na were 0.2, 0.64 and 1.08 g Na/L, respectively. To minimize water loss and inhibit algae growth, all Erlenmeyer flasks were covered with aluminum foil, followed by incubation in a growth chamber (illumination intensity: 20,000 lux; relative humidity: 60 ± 2%). Four independent biological replicates were deployed at each Cd-/Na-/Cd+Na-treated concentration in the 3-d experiment. Each independent biological replicate contained ten rice seedlings of similar size. The selection criteria for exposure concentrations of Cd and Na are presented in Section 2.5 and **Supporting information M1**.

### 2.2 Effects of temperature and atmospheric CO_2_ levels

A CO_2_ controlled environment chamber (CCEC, LRH-325-GSIE-T) was obtained from Shaoguan Taihong Medical Appliance Co., Ltd. Three different levels of CO_2_ at 360 (ambient), 450 (medium) and 700 ppm (high) were designed. The testing temperature was set at either 23°C or 27°C. The relative humidity in the CCEC was 60 ± 2%. Liquid CO_2_ was stored in a tank connected to the computer-controlled commercial CO_2_ injection system with a PID controller that served as the CO_2_ source. The discharge of CO_2_ in the CCEC was manually regulated by a needle valve that was connected to mechanical flow meters. The CO_2_ concentration in the CCEC was a real-time detection and recorded by a computer. The resolution of CO_2_ levels in this CCEC is 1 ppm. Four treatments and their abbreviations were as follows:

***Control A* (T1 + ambient CO_2_)**: rice seedlings were exposed to Solution A, Solution B and Solution C for 3 days and incubated in CCEC with a level of CO_2_ at 360 ppm (ambient CO_2_) and a temperature of 23°C;

***Treatment 1A* (T1 + medium CO_2_)**: rice seedlings were exposed to Solution A, Solution B and Solution C for 3 days and incubated in CCEC with a level of CO_2_ at 450 ppm (medium CO_2_) and a temperature of 23°C;

***Treatment 2A* (T1 + high CO_2_)**: rice seedlings were exposed to Solution A, Solution B and Solution C for 3 days and incubated in CCEC with a level of CO_2_ at 700 ppm (high CO_2_) and a temperature of 23°C.

***Control B* (T2 + ambient CO_2_)**: rice seedlings were exposed to Solution A, Solution B and Solution C for 3 days and incubated in CCEC with a level of CO_2_ at 360 ppm (ambient CO_2_) and temperature at 27°C;

***Treatment 1B* (T2 + medium CO_2_)**: rice seedlings were exposed to Solution A, Solution B and Solution C for 3 days and incubated in CCEC with a level of CO_2_ at 450 ppm (medium CO_2_) and a temperature of 27°C;

***Treatment 2B* (T2 + high CO_2_)**: rice seedlings were exposed to Solution A, Solution B and Solution C for 3 days and incubated in CCEC with a level of CO_2_ at 700 ppm (high CO_2_) and a temperature of 27°C.

### 2.3 Relative growth rate

The relative growth rate (RGR) is one of the most important parameters reflecting the overall performance of plant physiology under environmental abuse (Ling et al., 2021). The method for calculating the RGR was identical to our previous study (Lin et al., 2020) and was based on the following equation:


(1) $$RGR=\frac{W_{\left(F\right)}-W_{\left(I\right)}}{W_{\left(I\right)}}\times 100\%$$

where *W*_(I)_ and *W*_(F)_ are the initial and final fresh weights of rice seedlings, respectively.

### 2.4 Inhibition rate

The inhibition rate (IR, %) of RGR was calculated using the following equation:


(2) $$IR_{\left(C,t\right)}=\left(1-\frac{1/n\sum_{i-=1}^{n}RGR_{\left(C,t\right)}}{1/m\sum_{j=1}^{m}RGR_{\left(0,t\right)}}\right)\times 100$$

where *C* is the concentration of Cd and Na, *t* is the exposure period (d), *RGR* is the relative growth rate, *i* is replicate 1, 2,…, *n* and *j* is control 1, 2,…, *m*.

### 2.5 Determination of effective concentration

The effective concentration (EC) is the dose of pollutants that resulted in inhibitory effects on the possible parameters selected. In this study, the IR of RGR was used as a sensitive endpoint to estimate the EC value for Cd and Na treatments. The nominal concentrations of Cd treatments were 0, 0.25, 0.50, 1.00, 2.00, 4.00 and 8.00 mg Cd/L, while the nominal concentrations of Na treatments were 0, 0.1, 0.3, 0.5, 0.9, 1.2, and 1.6 g Na/L. For estimation of EC values, initial concentrations of Cd and Na were log_10_ transformed. Linear regression between log_10_Cd or Log_10_Na and IR (%) was plotted to calculate the concentrations of EC_20_, EC_50_ and EC_75_, which refer to the inhibition of 20%, 50%, and 75% of the RGR at 360 ppm CO_2_ and 25°C in comparison to the control, which were used as nominal initial concentrations for the tests with varying temperature and CO_2_ concentrations. More detailed information is presented in **Supporting information M1**.

### 2.6 Measurement of Ca and Na in rice seedlings

After 3 d of exposure, rice seedlings were collected and rinsed with distilled water and placed in a pretreated solution containing MES-Tris buffer (pH=6.0) for 4 h to remove all additional ions from the root surface and from the apparent free space. Then, rice seedlings were divided into roots and shoots. The remaining procedure was identical to our previous work (Fan et al., 2020). Dried plant tissues were digested with HNO_3_-HClO_4_ (v/v: 4:1) solution. The content of total Cd and Na in rice tissues from different treatments was determined by using inductively coupled plasma-atomic emission spectrometry (ICP−AES, PerkinElmer Optima 700 DV).

### 2.7 Data analysis

All data obtained were subjected to analysis of variance (ANOVA) and Tukey’s multiple range test at a significance level α of 0.01 or 0.05 between the treatments and control (**Supporting information M1**). The average of four independent biological replications ± the standard deviations (vertical lines) was used. Values within each graph followed by different letter(s) are significantly different (*P*< 0.05). The significance of correlations was judged using tabled values for critical *r* (significance level α was 0.01 or 0.05).

## 3 Results

### 3.1 Effects on plant growth

**Figure 1:**
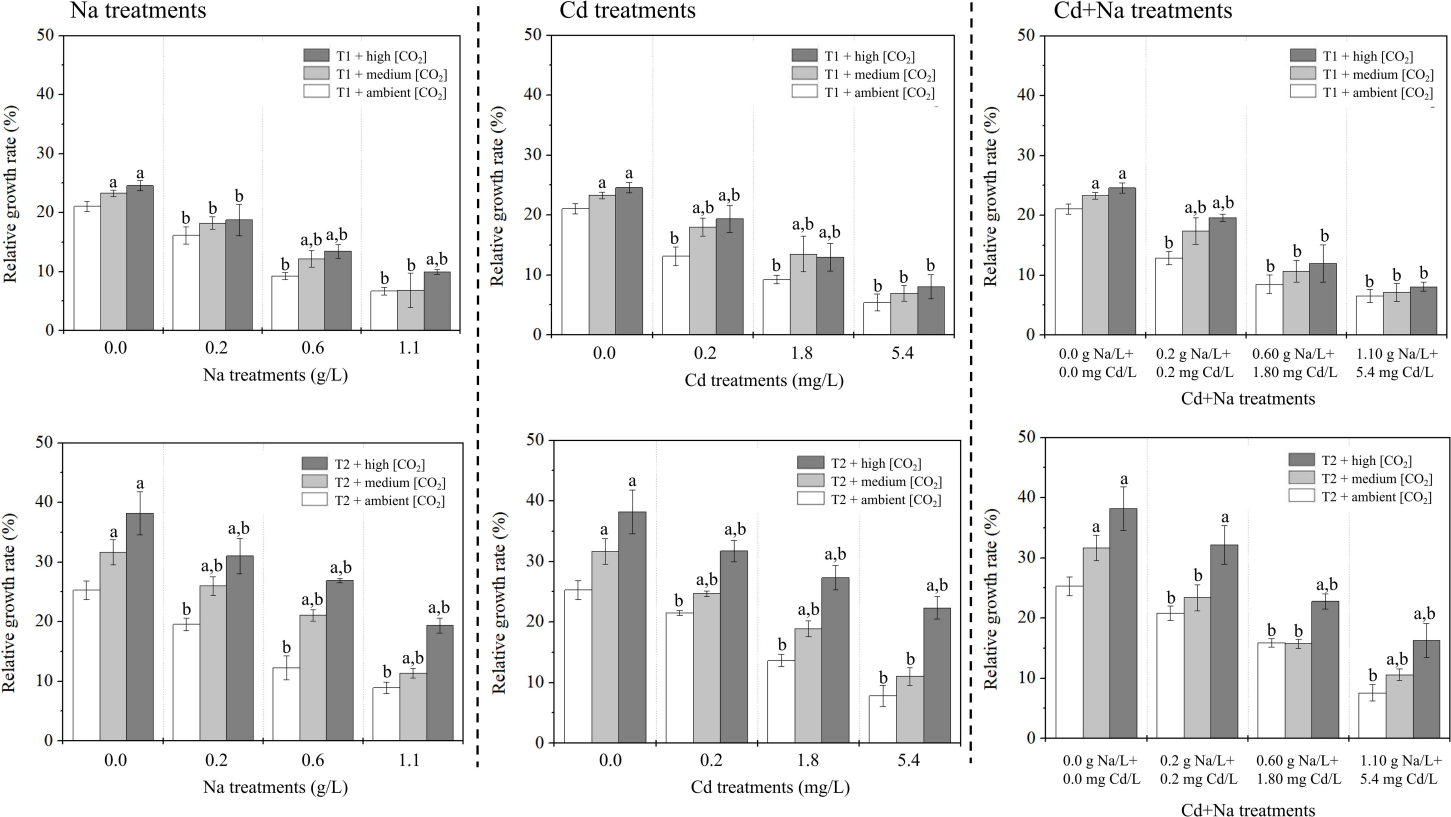
Relative growth rate (%) of rice seedlings. Left: Na treatments (0, 0.2, 0.6 and 1.1 g Na/L); middle: Cd treatments 0, 0.2, 1.8, 5.4 mg Cd/L); right; Na + Cd treatments. Top: T1 (23°C); bottom: T2 (27°C). The lowercase letter “a” indicates a significant difference between high/medium CO_2_ and ambient CO_2_ at the same temperature. The lowercase letter “b” indicates a significant difference between the Cd (i.e., 0.2, 1.8, and 5.4 mg Cd/L), Na (i.e., 0.2, 0.6, and 1.1 g Na/L), Cd+Na (i.e., 0.2 mg Cd/L+ 0.2 g Na/L, 1.8 mg Cd/L+ 0.6 g Na/L, and 5.4 mg Cd/L+ 1.1 g Na/L) treatments and the 0.0 mg/L treatment.

The measured RGRs for the Na treatments (0, 0.2, 0.6 and 1.1 g/L), Cd treatments (0, 0.2, 1.8, 5.4 mg/L), and Na + Cd treatments at T1 (23°C) and T2 (27°C) are shown in **Figure 1**. Under Na stress and T1 (T2), the RGRs of both ambient/medium/high CO_2_-treated rice seedlings ranged between 6.69-21.07%, 6.85-23.27%, and 9.94-24.58% (8.91-25.29%, 11.35-31.68%, and 19.38-38.23%), respectively. Under Cd stress and T1 (T2), the RGRs of both ambient/medium/high CO_2_-treated rice seedlings ranged between 5.43-21.07%, 6.97-23.27%, and 8.06-24.58% (7.80-25.29%, 11.03-31.68%, and 22.34-38.23%), respectively. Under Na+Cd stress and T1 (T2), the RGRs of both ambient/medium/high CO_2_-treated rice seedlings ranged between 6.55-21.07%, 7.14-23.27%, and 8.09-24.58% (7.58-25.29%, 10.58-31.68%, and 16.26-38.23%), respectively. Apparently, for all treatments, the growth is positive, i.e., RGR is positive. In all cases, growth was significantly better at 27°C (*P*< 0.05) and at higher CO_2_. Growth inhibition of (approximately) 25%, 50% and 75% was observed for salt treatments with Na at 0.2, 0.6 and 1.1 g/L, respectively, and with Cd at 0.2, 1.8, and 5.4 mg/L, respectively. At 23°C, the effect of Na and Cd applied simultaneously was not significantly different from that of individual treatments. At 27°C, a slight depression of growth can be noticed in the combined treatment in relation to the single treatment with Na or Cd only.

### 3.2 Measured concentration of Cd in rice at different temperatures and CO_2_ concentrations

**Figure 2:**
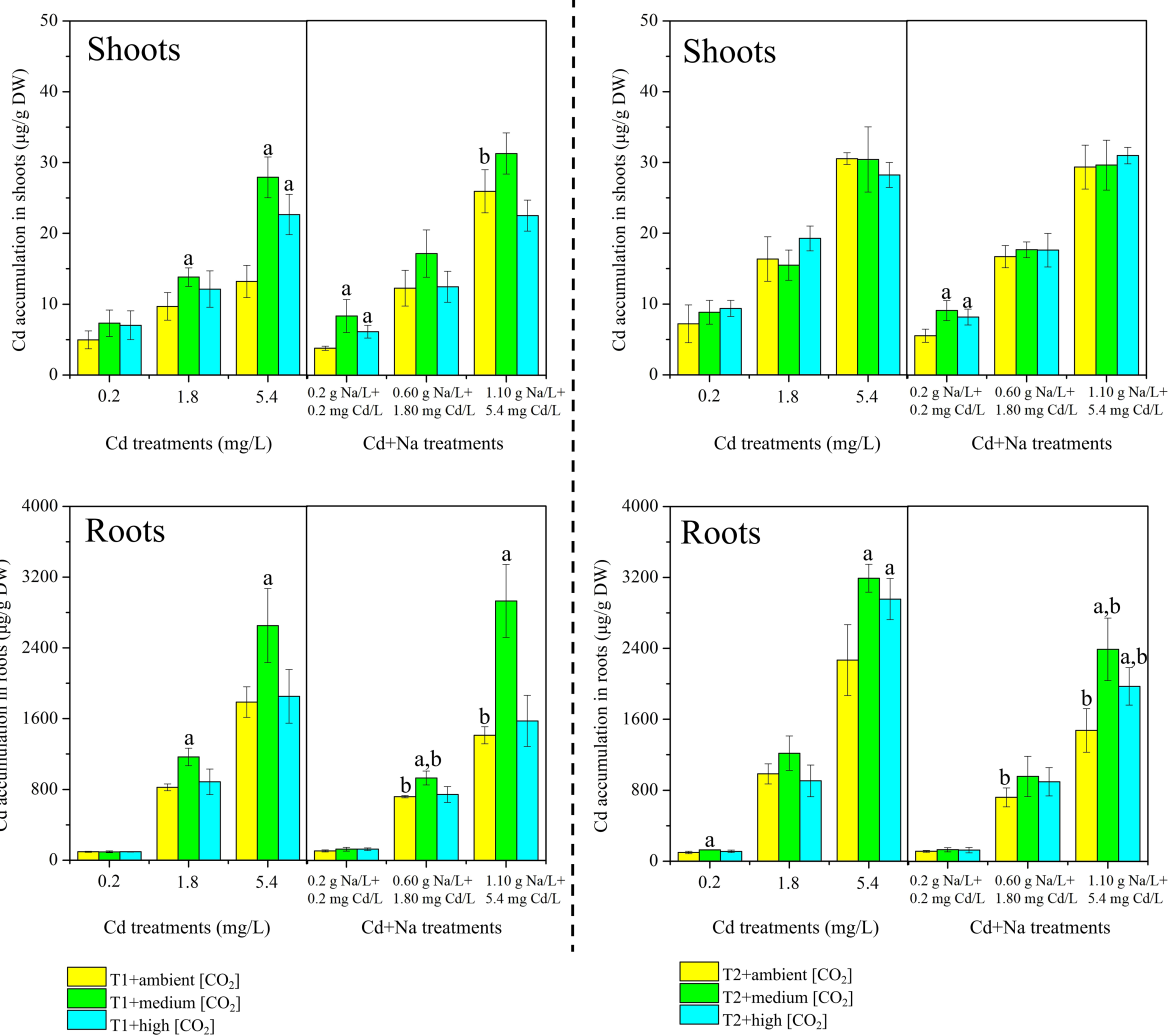
Measured concentrations of cadmium (Cd, µg/g dry weight) in rice plants. Top: shoots; bottom: roots. Left side: T1 (23°C); right side: T2 (27°C). The lowercase letter "a" indicates a significant difference between high/medium CO_2_ and ambient CO_2_ at the same temperature. The lowercase letter "b" indicates a significant difference between Cd treatments and Cd+Na treatments under the same temperature and CO_2_ conditions.

The measured Cd content in rice shoots and roots separated for T1 (23°C) and T2 (27°C) is shown in **Figure 2**. Under T1 and ambient CO_2_ treatments, the concentrations of Cd in rice shoots (roots) with Cd stress ranged between 4.97-13.21 μg/g DW (95.31-1787.23 μg/g DW), while the concentrations of Cd in rice shoots (roots) with Na+Cd stress ranged between 3.80-25.93 μg/g DW (107.5-1411.33 μg/g DW). Under T1 and medium CO_2_ treatments, the concentrations of Cd in rice shoots (roots) with Cd stress ranged between 7.31-27.66 μg/g DW (94.46-2652.8 μg/g DW), while the concentrations of Cd in rice shoots (roots) with Na+Cd stress ranged between 8.35-31.25 μg/g DW (126.5-2928.09 μg/g DW). Under the T1 and high CO_2_ treatments, the concentrations of Cd in rice shoots (roots) with Cd stress ranged between 7.02-22.67 μg/g DW (95.15-1852.19 μg/g DW), while the concentrations of Cd in rice shoots (roots) with Na+Cd stress ranged between 6.14-22.50 μg/g DW (126.77-1574.02 μg/g DW).

Under T2 and ambient CO_2_ treatments, the concentrations of Cd in rice shoots (roots) with Cd stress ranged between 7.21-30.55 μg/g DW (97.81-2267.69 μg/g DW), while the concentrations of Cd in rice shoots (roots) with Na+Cd stress ranged between 5.55-29.33 μg/g DW (113.65-1474.37 μg/g DW). Under T2 and medium CO_2_ treatments, the concentrations of Cd in rice shoots (roots) with Cd stress ranged between 8.84-30.43 μg/g DW (127.72-3191.39 μg/g DW), while the concentrations of Cd in rice shoots (roots) with Na+Cd stress ranged between 9.09-29.61 μg/g DW (131.20-2387.40 μg/g DW). Under T2 and high CO_2_ treatments, the concentrations of Cd in rice shoots (roots) with Cd stress ranged between 9.38-28.23 μg/g DW (110.39-2955.65 μg/g DW), while the concentrations of Cd in rice shoots (roots) with Na+Cd stress ranged between 8.18-30.95 μg/g DW (126.13-1970.16 μg/g DW).

Notably, the concentration of Cd in shoots was much lower than that in roots. In shoots, higher concentrations were observed only at 27°C (*P*< 0.05). At 23°C, there is a trend toward high Cd concentrations in shoots when coexposed to Na and at low and medium CO_2_ (360 and 450 ppm), but this is not seen at 700 ppm CO_2_ and not at 27°C. At 23°C, the highest Cd concentration is found at 450 ppm CO_2_. Again, this trend is not apparent at 27°C, where concentrations of Cd are rather equal for all levels of CO_2_. As growth is best at 700 ppm CO_2_ and at 27°C, it can be concluded that the total uptake (mass of Cd) must be highest for these conditions.

Additionally, for roots, at 23°C, the highest concentrations of Cd were found with 450 ppm CO_2_, and similar concentrations were found for 360 and 700 ppm CO_2_. The trend is similar for 27°C but less pronounced, and concentrations are highest at 450 ppm CO_2_ and lowest at 360 ppm CO_2_. Concentrations of Cd in roots are higher when exposed solely to Cd than when exposed to Cd+Na stress.

### 3.3 Measured concentration of Na in rice at different temperatures and CO_2_ concentrations

**Figure 3:**
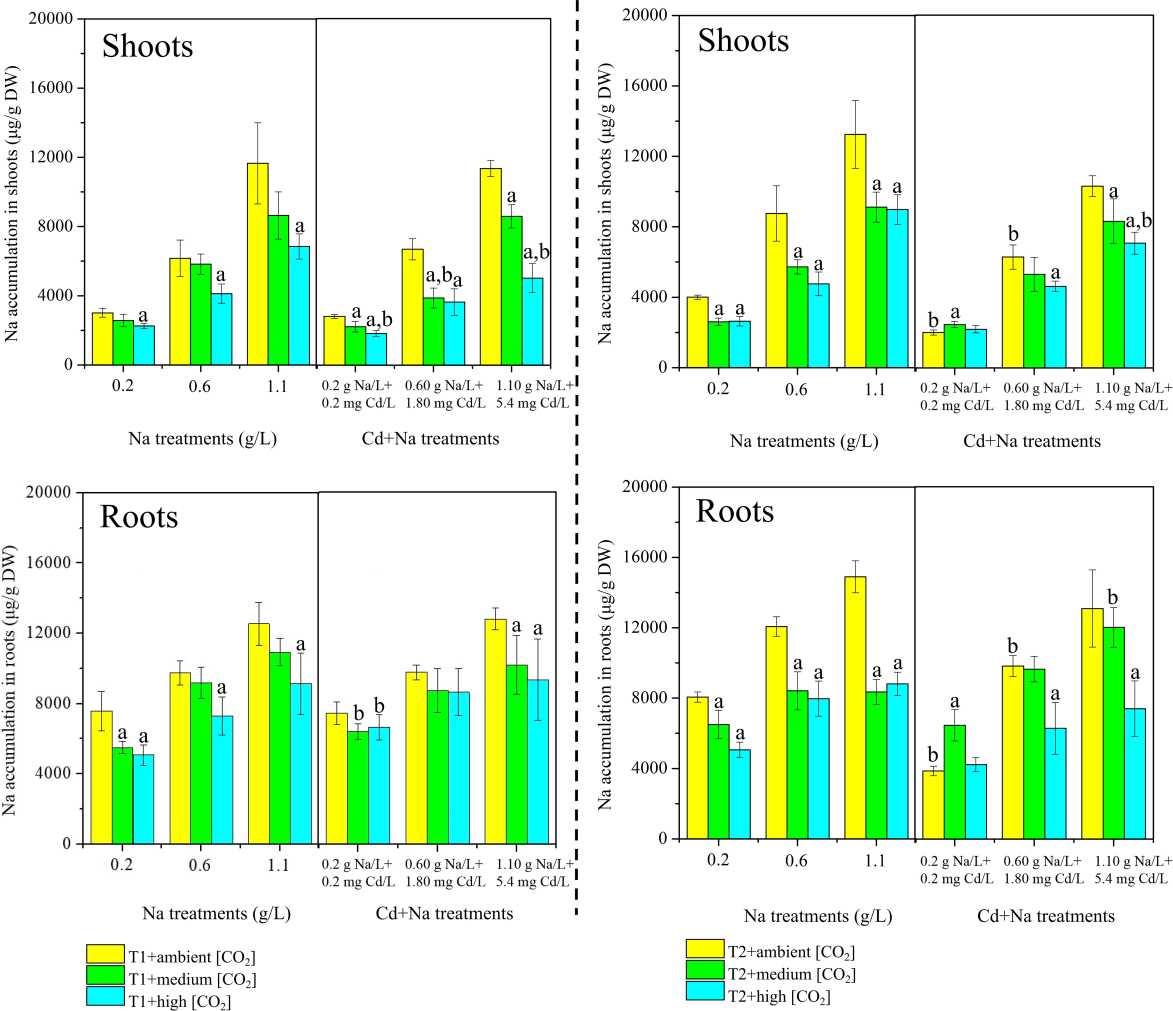
Measured concentrations of sodium (Na, µg/g dry weight) in rice plants. Top: shoots; bottom: roots. Left side: T1 (23°C); right side: T2 (27°C). The lowercase letter "a" indicates a significant difference between high/medium CO_2_ and ambient CO_2_ at the same temperature. The lowercase letter "b" indicates a significant difference between the Na treatments and Cd+Na treatments under the same temperature and CO_2_ conditions.

The measured concentrations of Na in rice shoots and roots separated for T1 (23°C) and T2 (27°C) are shown in **Figure 3**. Under T1 and ambient CO_2_ treatments, the concentrations of Na in rice shoots (roots) with Na stress ranged between 3011.61-11655.06 μg/g DW (7569.71-12545.83 μg/g DW), while the concentrations of Na in rice shoots (roots) with Na+Cd stress ranged between 2813.5-11340.46 μg/g DW (7453.15-12805.63 μg/g DW). Under T1 and medium CO_2_ treatments, the concentrations of Na in rice shoots (roots) with Na stress ranged between 2568.85-8641.81 μg/g DW (5500.33-10866.99 μg/g DW), while the concentrations of Na in rice shoots (roots) with Na+Cd stress ranged between 2220.49-8589.50 μg/g DW (6425.30-10252.57 μg/g DW). Under T1 and high CO_2_ treatments, the concentrations of Na in rice shoots (roots) with Na stress ranged between 2257.15-6848.62 μg/g DW (5067.89-9131.93 μg/g DW), while the concentrations of Na in rice shoots (roots) with Na+Cd stress ranged between 1825.34-5020.30 μg/g DW (6657.62-9354.65 μg/g DW).

Under T1 and ambient CO_2_ treatments, the concentrations of Na in rice shoots (roots) with Na stress ranged between 4004.82-13243.01 μg/g DW (8057.37-14898.06 μg/g DW), while the concentrations of Na in rice shoots (roots) with Na+Cd stress ranged between 2006.02-10301.83 μg/g DW (3867.43-13083.43 μg/g DW). Under T1 and medium CO_2_ treatments, the concentrations of Na in rice shoots (roots) with Na stress ranged between 2607.55-9115.69 μg/g DW (6500.54-8351.60 μg/g DW), while the concentrations of Na in rice shoots (roots) with Na+Cd stress ranged between 2457.76-8309.92 μg/g DW (6459.82-12013.72 μg/g DW). Under T1 and high CO_2_ treatments, the concentrations of Na in rice shoots (roots) with Na stress ranged between 2635.73-8979.19 μg/g DW (5062.06-8811.56 μg/g DW), while the concentrations of Na in rice shoots (roots) with Na+Cd stress ranged between 2187.13-7068.07 μg/g DW (4223.16-7396.10 μg/g DW).

Overall, Na concentrations in roots are higher than in shoots, but the difference is small. The maximum concentrations in roots reached 15 mg/g DW, and those in shoots were >12 mg/g DW. There is little difference in concentrations with/without additional Cd. There is also little difference between concentrations at T1 (23°C) and T2 (27°C). However, for almost all variants, Na concentrations have a strong tendency to be lower at high CO_2_ levels.

### 3.4 Translocation factors of Cd and Na in rice seedlings

**Figure 4:**
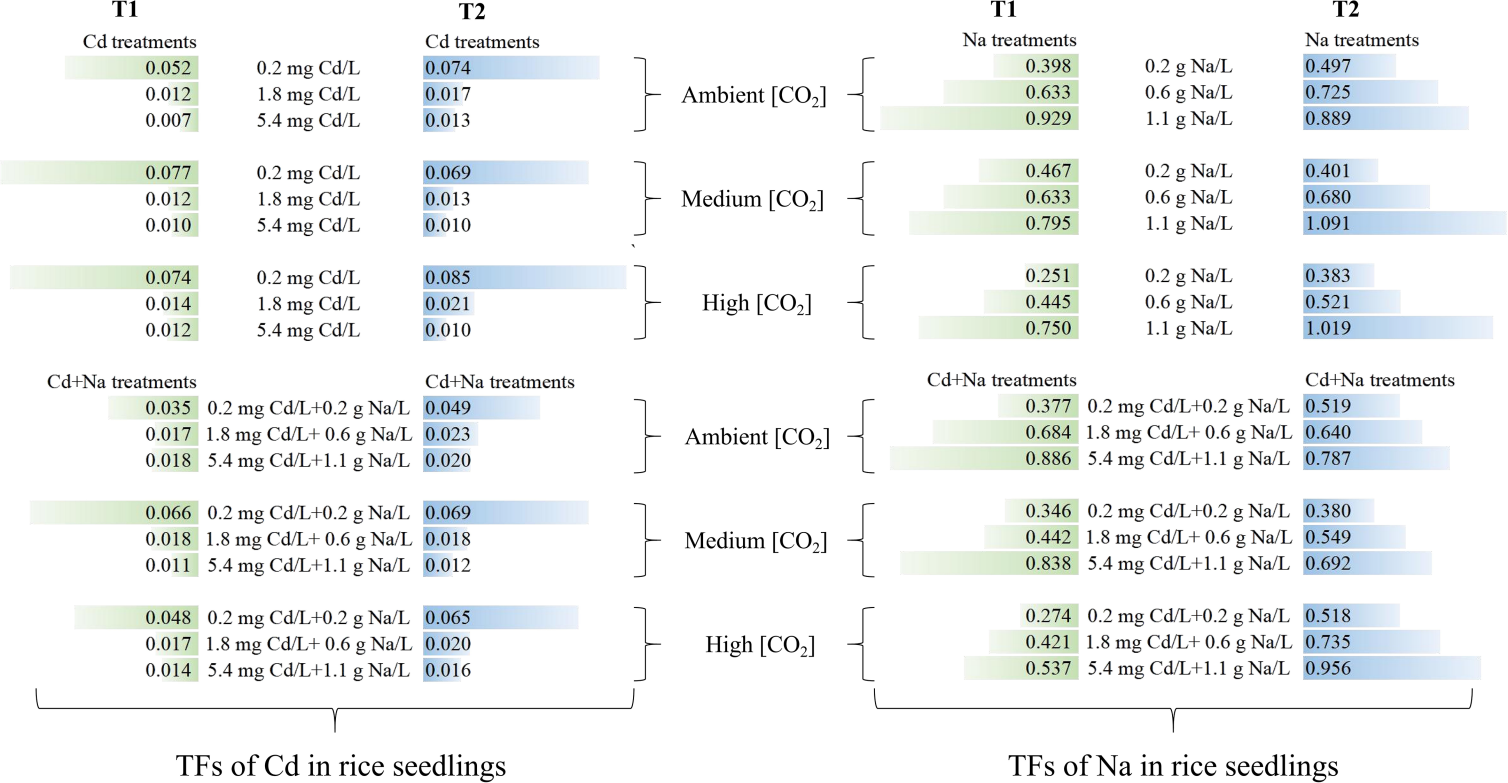
Translocation factors of Cd and Na under different temperature and CO_2_ conditions.

The effect of temperature and CO_2_ on the translocation factors (TFs) of Cd and Na in rice seedlings is shown in **Figure 4**. Apparently, the TFs of Cd in rice seedlings decrease with increasing Cd and Cd+Na concentrations. In contrast, the TFs of Na in rice seedlings decreased with increasing Cd and Cd+Na concentrations. Compared with T1 (23°C) at the same CO_2_ concentrations, T2 (27°C) promotes the translocation of Cd and Na in rice seedlings.

In addition, at 23°C, medium and high CO_2_ generally promoted Cd translocation in rice seedlings under 0.2 mg Cd/L stress, but no significant effect under 1.8 and 5.4 mg Cd/L stress was observed. While medium and high CO_2_ have little effect on Na translocation in rice seedlings under 0.2 and 0.6 g Na/L stress, high CO_2_ inhibits Na translocation under 1.1 g Na/L stress. At 27°C, high CO_2_ had an apparent effect on Cd translocation in rice seedlings under 0.2, 0.6, and 1.1 g Na/L stress. While medium CO_2_ inhibits Na translocation in rice seedlings under 0.2, 0.6, and 1.1 g Na/L stress, high CO_2_ promotes Na translocation.

### 3.5 Coexposure to Na and Cd

**Figure 5:**
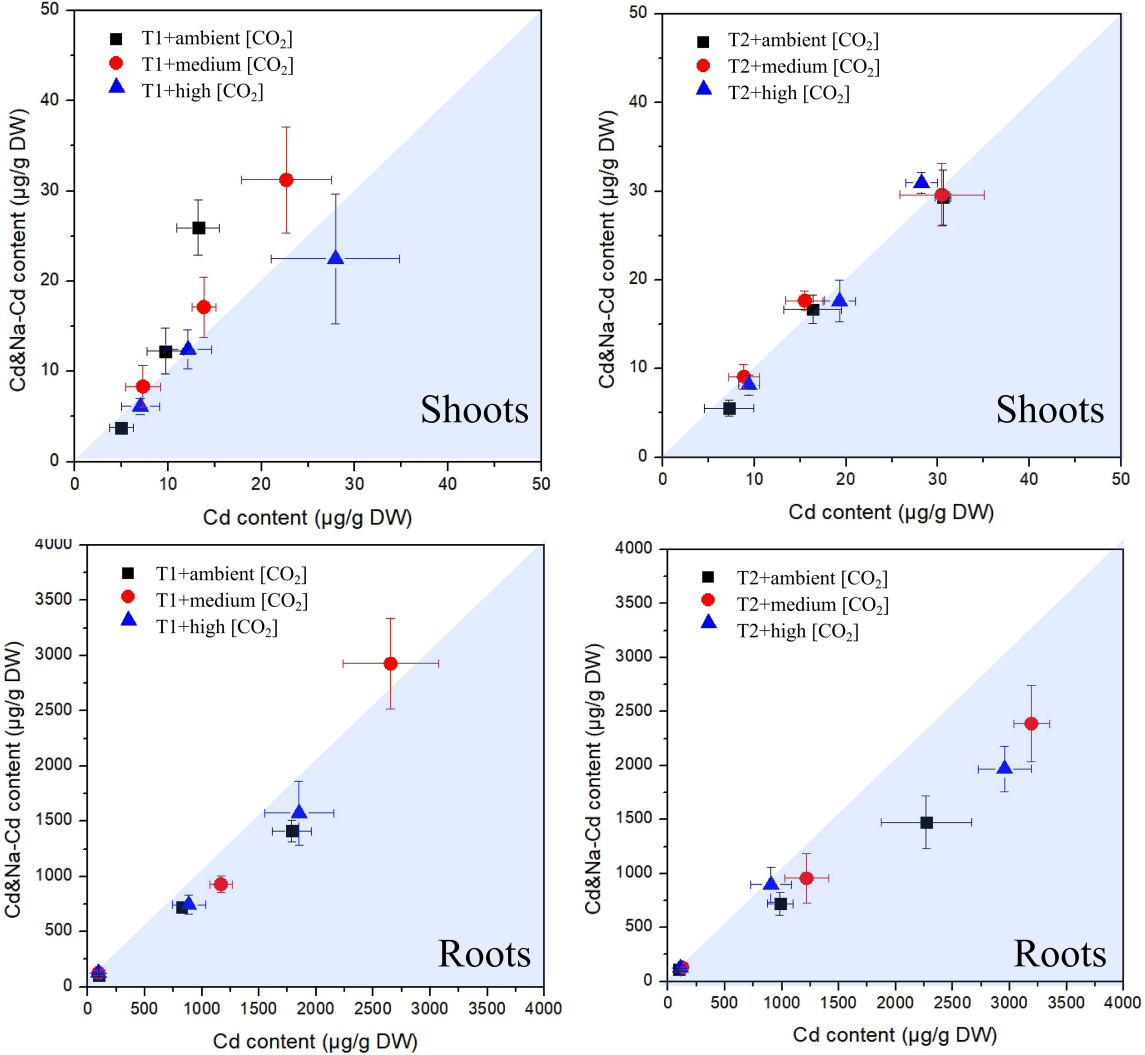
Plot of measured cadmium concentrations in plants exposed to Cd+Na (Y-axis) versus those measured in plants exposed solely to Cd (X-axis); top: shoots; bottom: roots; left side: T1 (23°C); right side: T2 (27°C).

The measured concentrations of Cd in plants exposed to Cd+Na versus those measured in plants exposed solely to Cd are shown in **Figure 5**. This underlines what can be seen in **Figure 2**, that is, the concentrations of Cd in shoots are generally rather similar. At 23°C, there is a tendency for higher Cd in shoots when additionally exposed to Na, but this trend almost vanishes at 27°C. In contrast, for roots, there was a tendency for higher Cd concentrations when solely exposed to Cd, and the deviation between the two exposure sets was stronger at 27°C. An influence of CO_2_ is not apparent.

**Figure 6:**
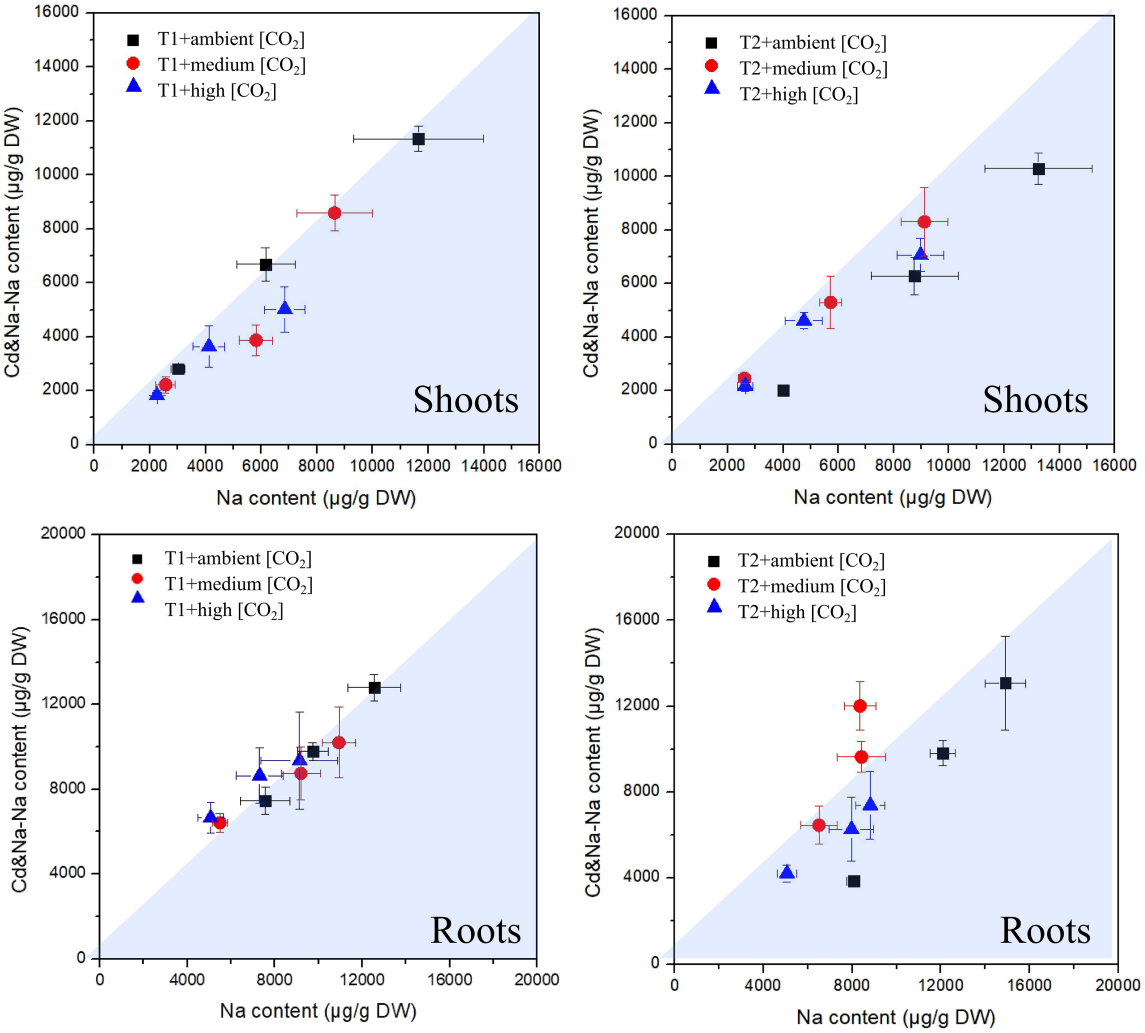
Plot of measured sodium concentrations in plants exposed to Cd+Na (Y-axis) versus those measured in plants exposed solely to Na (X-axis); top: shoots; bottom: roots; left side: T1 (23°C); right side: T2 (27°C).

The measured concentrations of Na in plants exposed to Cd+Na versus those measured in plants exposed solely to Na are shown in **Figure 6**. There is a small tendency for higher concentrations of Na in shoots and roots at 27°C, while at T1, the difference vanishes. Concentrations are highest at low CO_2_, but this is the case for both types of exposure (with/without Cd). Overall, both concentrations of Cd and Na in roots and shoots are surprisingly similar, almost independent from the single or combined exposure.

## 4 Discussion

### 4.1 The growth of stressed plants improved by higher CO_2_ and temperature

Higher temperatures may lead to an increase in the agricultural productivity of plants when the plants are grown in a suboptimal temperature range (Berry and Bjorkman, 1980; IPCC, 2019), and elevated CO_2_ in air increases water use efficiency and stimulates the growth of plants (Ainsworth and Rogers, 2007; IPCC, 2019). In general, increases in CO_2_ can immediately stimulate photosynthesis and enhance the carbohydrate content, particularly in C_3_ plants of rice (Wang et al., 2016). The interactive effects of temperature and CO_2_ on the physiology, growth and development of various plant species have also been studied (Wang et al., 2012). However, very few studies control four parameters (i.e., temperature, CO_2_, Cd, and Na) simultaneously; thus, the findings presented here are quite unique. Cd and Na (or salt) are two toxic chemicals affecting plant growth and development due to their toxicity (Das et al., 1997; Zhu, 2001; Singh et al., 2013; Haider et al., 2021). In the present study, we noticed that individual or combined treatments of Cd and Na significantly decreased the RGR of rice seedlings, but the synergistic or antagonistic effects of Cd and Na seem not significant, particularly at T1 (23°C) or high CO_2_. In addition, the T2 (27°C) and medium/high CO_2_ levels significantly increased the RGR of stressed plants compared to the T1 (23°C) and ambient CO_2_ levels. Therefore, we believe that increasing temperature can stimulate the activities of enzymes in plants, such as RuBisCO (Suganami et al., 2021), and elevated CO_2_ can improve the carboxylation capacity of RuBisCO in plants, which can be converted into nonstructural carbohydrates and some detoxicants under ionic stress conditions (Bazinet et al., 2022), thereby reducing phytotoxicity and promoting plant growth.

Several studies have examined the effect of elevated CO_2_ on the toxicity of Na or salt to plants. These studies revealed that the salt tolerance of plants increases at elevated CO_2_, while negative effects have not been observed (Pérez-López et al., 2009; Geissler et al., 2015; Yu et al., 2015). The effect of CO_2_ on the uptake of Na by plants occurs in addition to that of increased temperatures. Eller et al. (2014) studied the mutual effects of elevated temperature and CO_2_ on the tolerance of plant species towards soil salinity and found that a temperature increase of 5°C and 700 ppm CO_2_ alleviated salt stress in two phenotypes of *Phragmites*. The contribution of each factor was not investigated.

### 4.2 Effects of temperature and CO_2_ on the distribution and translocation of Cd and Na

It is evident that exposed rice contained larger quantities of Cd in roots than in shoots (**Figure 2**), while the content of Na in rice shoots was rather similar to that in roots (**Figure 3**). Moreover, the individual and mutual effects of temperature and CO_2_ on the distribution and translocation of Cd and Na in rice seedlings were not identical.

#### 4.2.1 Temperature effects at the same CO_2_ level

Similar to evaporation from soil, the transpiration of water from plant leaves increases with higher temperatures. This leads to a more rapid transport of solutes into and through plants. Additionally, enzymatic processes accelerate with temperature (Arrhenius Law). In this study, we noticed that the content of Cd in rice roots and shoots under Cd stress increased with increasing temperature in all CO_2_ treatments. However, ample studies have shown that the temperature-mediated uptake and translocation of Cd and Na in plants are different. Herein, we noticed that increasing temperature promoted the uptake and accumulation of Na in rice tissues under Na stress at high CO_2_ while inhibiting the uptake and accumulation of Na at medium/ambient CO_2_. Unlike for Cd, the cell wall cannot act as a barrier to Na uptake, and uptake of Na from environmental media into roots is passive and follows the transpiration stream (Hussain et al., 2017), with subsequent active (enzymatic) exclusion (Trapp et al., 2008). In contrast, the degree to which temperature can affect Cd accumulation depends on the chemistry of the root zone, plant growth, and biomass (Fritioff et al., 2005; Peer et al., 2005). Root immobilization can elucidate substantial amounts of accumulated metals in plants and is affected by temperature, which can modify the structure and function of cell walls by inducing the synthesis of plant hormones (Liu et al., 2019; Cheng et al., 2020). In line with this, we found that the root-to-shoot TFs of Cd under T2 (27°C) were generally higher than those under T1 (23°C). Ge et al. (2016) also found an enhanced translocation of Cd from roots to shoots at elevated temperatures for rice.

#### 4.2.2 CO_2_ effects at the same temperature

The responses of Cd and Na in rice tissues to varying CO_2_ concentrations in air were different. The concentration of Cd in rice tissues and, in particular, in roots increased at 450 ppm CO_2_ but decreased at 700 ppm CO_2_. The concentration of Na in rice tissues significantly decreased with increasing CO_2_ concentration and was lowest at 700 ppm CO_2_. This suggests that the uptake and translocation of Cd and Na are affected differently by the variation in CO_2_. Indeed, elevated CO_2_ resulting in higher Cd concentrations in plant tissues has been observed in different plant species (Li et al., 2010; Guo et al., 2011; Guo et al., 2015). Tang et al. (2003) and Wu et al. (2009) demonstrated that elevated CO_2_ might lower the pH of the rhizospheric environment, which leads to a decline in the K_d_ of Cd and other heavy metals (Sheppard, 2011) and thereby promotes the plant uptake of heavy metals. In addition, the influence of elevated CO_2_ on cell wall properties might explain this finding. Genes encoding expansins, esterase, and xyloglucan endotransglycosylase, which play important roles in cell wall loosening, are upregulated at elevated CO_2_ and thus facilitate plant tissue expansion (Gamage et al., 2018). Plant hormones also play a major role in accumulating Cd in plants at elevated CO_2_ (Wei et al., 2013) because plant hormones such as ethylene, ausins, gibberellic acids, and cytokinins can synergistically regulate cell division and expansion and control root and shoot elongation (Seneweera et al., 2003; Cato et al., 2013), thereby providing a larger zone for Cd storage in plants. The expression of metal tolerance protein genes in plants, such as the MTP family, may be activated by elevated CO_2_, enhancing their metal tolerance (El-Sappah et al., 2021a; El-Sappah et al., 2021b). On the other hand, a CO_2_-stimulated increase in plant biomass may lead to dilution and thus lower tissue concentrations of Cd and other heavy metals despite increasing uptake (Duval et al., 2011). Therefore, the mechanisms involved in CO_2_-mediated Cd uptake and translocation in plants should be further investigated by using physio-biochemical analysis and multiomics approaches.

Transpiration and thus stomatal conductance are some of the most important factors affecting the uptake and translocation of Cd and Na in plants (Bauer-Gottwein et al., 2008; Trapp et al., 2008). Herein, we noticed that high CO_2_ inhibited the translocation of Cd and Na in rice seedlings. Based on the reports by Ainsworth and Long (2005) and Leakey et al. (2009), the stomatal conductance of plant leaves decreased under elevated CO_2_. Moreover, elevated CO_2_ increases outwards rectifying K^+^ channels relative to inwards rectifying K^+^ channels, resulting in stomatal closure (Brearley et al., 1997). Apparently, stomatal closure directly reduced the transpiration stream of rice seedlings, eventually inhibiting Na uptake and translocation. Additionally, a previous study suggested that elevated CO_2_ reduced transpiration by 30%, which not only affected root element acquisition by reducing mass flow into plants but also diminished the accumulation of elements in shoots through the translocation of sylem sap. Another study confirmed that elevated CO_2_-mediated impairment of element translocation into the shoots is related to a specific effect on xylem morphogenesis (Cohen et al., 2019).

### 4.3 Effects of a mixture of Cd and Na on rice seedlings

To evaluate the effect of mixtures of Cd and Na on rice seedlings under different temperature and CO_2_ conditions, we determined the Cd and Na contents in rice tissues under coexposure to Cd and Na. In general, the content of Cd in rice tissues under Cd+Na stress was higher than that under Cd stress under T1+medium CO_2_ conditions, while the content of Cd in rice tissues under Cd+Na stress was lower than that under Cd stress under T2+ambient/medium/high CO_2_ conditions. In addition, the content of Na in rice tissues under Cd+Na stress was generally lower than that under Na stress under T1(T2)+ambient/medium/high CO_2_ conditions. Interestingly, the interaction was not very pronounced but observable. These results implied that the temperature should be considered when CO_2_ is used as the regulator in the phytoremediation of Cd/Na/Cd+Na pollution. In addition, we noticed that the concentration of Cd in roots was depressed by coexposure to Na and more at T2, while with T1, more Cd was translocated into shoots. These changes indicate that the uptake processes of Cd and Na in rice are mostly independent, and only minor interactive effects occurred during exposure to mixtures of Cd and Na. A plausible explanation is that both molecules have very different radii and different charges (Na^+^ versus Cd^2+^), and it is unlikely that the same transporter enzymes are involved. However, Cd and Na are both cations and might compete for electrically charged adsorption sites. This may be the mechanism behind the lower Cd concentration in roots when the Na concentration is high. The cytosolic ionic strength was 0.3 M, in xylem sap at 0.01 (Sitte et al., 1991). The highest measured concentrations of Na (15.0 g/L) correspond to a molality of 0.6 M. According to the Debye-Hückel theory (Debye and Hückel, 1923), the chemical activity of a bivariate ion decreases drastically at such an ionic strength, which can explain the elevated concentrations of Cd in roots and the stimulated transport of Cd into shoots in the presence of Na. Hence, the effects of Na on the uptake of Cd may be attributed to physico-chemical processes. This is less the case the other way round because molalities due to intracellular Cd concentrations are much smaller than those of Na. Generally, Cd shows a much higher concentration ratio of roots to external solution than Na and thus much higher adsorption. Overall, the effects of Cd in coexposure on Na uptake are also rather small and not very clear. The small changes in uptake when the mixture is applied may be among the reasons why the toxic effects are not more pronounced due to coexposure. It is likely that the modes of action of Na and Cd on rice seedlings are not the same and thus do not add (ECETOC, 2007).

## 5 Conclusions

This work clarifies the simultaneous effects of changing temperatures and CO_2_ concentrations on the mutual uptake, translocation and effects of Na and Cd on rice seedlings. The hydroponic experimental results presented here showed the individual and mutual effects of temperature and CO_2_ on Na and Cd tolerance of rice seedlings, judged by the biomass, Na/Cd distribution and translocation. The biomass response suggested that Cd and Na exposure significantly inhibited plant growth, but T2 and medium/high CO_2_ levels could alleviate the toxicity exerted by Cd and Na. Interestingly, the synergistic or antagonistic effects of Cd and Na were not significant, particularly at T1 or high CO_2_. The highest concentrations of Cd were observed at T2 and with 450 ppm CO_2_. Na accumulation in rice seedlings increased with temperature but was reduced by higher CO_2_. In summary, the optimum conditions to achieve low Cd concentrations in rice shoots are T1 and low or high CO_2_, while with respect to the toxic effects of Na and Cd alone or in mixture, they are T2 and high CO_2_. Another conclusion from the presented findings is that CO_2_ concentrations in laboratory studies with terrestrial plants have a strong impact on the results and should be monitored and reported. Further comprehensive studies are needed to clarify the possible tolerance mechanisms involved in RNA (e.g., mRNA, lncRNA, small RNA, and circRNA), proteins, and metabolites.

## Data availability statement

The original contributions presented in the study are included in the article/**Supplementary Material**. Further inquiries can be directed to the corresponding authors.

## Author contributions

Conceptualization, Methodology, Supervision, Writing-reviewing and editing, and Funding acquisition, ST and X-ZY. Writing-original draft preparation, and Visualization, Y-XF. Investigation, Data analysis, Visualization, PT, C-ZL, and QZ. All authors contributed to the article and approved the submitted version.
