# Supplementary material for: Individual and mutual effects of elevated carbon dioxide and temperature on salt and cadmium uptake and translocation by rice seedlings
Source: Front Plant Sci. 2023 Apr 5;14:1161334. doi: 10.3389/fpls.2023.1161334 (PMC10113512; doi:10.3389/fpls.2023.1161334)
Supplement: Supplementary file 1 [file DataSheet_1.doc]

***Supporting information for***

**The individual and mutual effects of elevated temperature and carbon dioxide on the salt and cadmium tolerance of rice seedlings**

Yu-Xi Feng^†^, Peng Tian^†^, Cheng-Zhi Li^†^, Qing Zhang^†^, Stefan Trapp^‡^,*, Xiao-Zhang Yu^†^,*

^†^College of Environmental Science & Engineering, Guilin University of Technology, Guilin, 541004, People’s Republic of China

^‡^ Department of Environmental Engineering, Technical University of Denmark, Bygningstorvet 115, 2800 Kongens Lyngby, Denmark

***Corresponding author**

Prof. Dr. Stefan Trapp. ORCID iD: [0000-0001-8968-6296](http://orcid.org/0000-0001-8968-6296)

Phone: [+4545251622](https://www.dtu.dk/english/service/phonebook/tel:+4545251622). E-mail: [sttr@env.dtu.dk](mailto: sttr@env.dtu.dk)

Prof. Dr. Xiao-Zhang Yu. ORCID iD: 0000-0001-7846-5017

Phone: +86 7735897016. E-mail: [xzyu@glut.edu.cn](mailto:xzyu@glut.edu.cn)

**Supporting information M1**

**2.5 Determination of effective concentrations**

Based on the equation (1) and (2), we determined the RGR and IR of rice seedlings under Cd treatments and Na treatments, respectively. The RGR and IR of rice plants from Cd and Na treatments were shown in **Table S1** and **Table** **S2**.

**Table S1** The RGR and IR rice plants from Cd treatments

| Conc. (mg Cd/L） | RGR (%) | S.D | IR (%) | S.D |
| --- | --- | --- | --- | --- |
| 0.00 | 21.2 | 3.73 | 0.00 | 6.94 |
| 0.25 | 16.4 | 1.02 | 22.5 | 4.84 |
| 0.50 | 13.9 | 0.44 | 34.3 | 2.07 |
| 1.00 | 11.9 | 3.17 | 43.7 | 14.98 |
| 2.00 | 9.15 | 2.19 | 56.8 | 10.35 |
| 4.00 | 8.36 | 1.04 | 60.5 | 4.92 |
| 8.00 | 3.60 | 0.75 | 83.0 | 3.56 |

**Table S2** The RGR and IR of rice plants from Na treatments

| Conc. (g Na/L) | RGR (%) | S.D | IR (%) | S.D |
| --- | --- | --- | --- | --- |
| 0.00 | 18.7 | 4.06 | 0.00 | 8.48 |
| 0.10 | 16.8 | 1.56 | 10.4 | 1.92 |
| 0.30 | 12.5 | 0.93 | 33.4 | 4.98 |
| 0.50 | 10.8 | 1.25 | 42.5 | 6.66 |
| 0.90 | 6.40 | 1.68 | 65.8 | 8.96 |
| 1.20 | 3.39 | 2.07 | 81.8 | 11.08 |
| 1.60 | 1.49 | 0.42 | 92.0 | 2.22 |

For estimation of EC values, nominal concentrations and IR of Cd (**Fig. S1**) and Na (**Fig. S2**) in rice plants were log_10_ transformed (The nominal concentrations of Cd treatments were 0, 0.25, 0.50, 1.00, 2.00, 4.00 and 8.00 mg Cd/L, while the nominal concentrations of Na treatments were 0, 0.1, 0.3, 0.5, 0.9, 1.2, and 1.6 g Na/L).


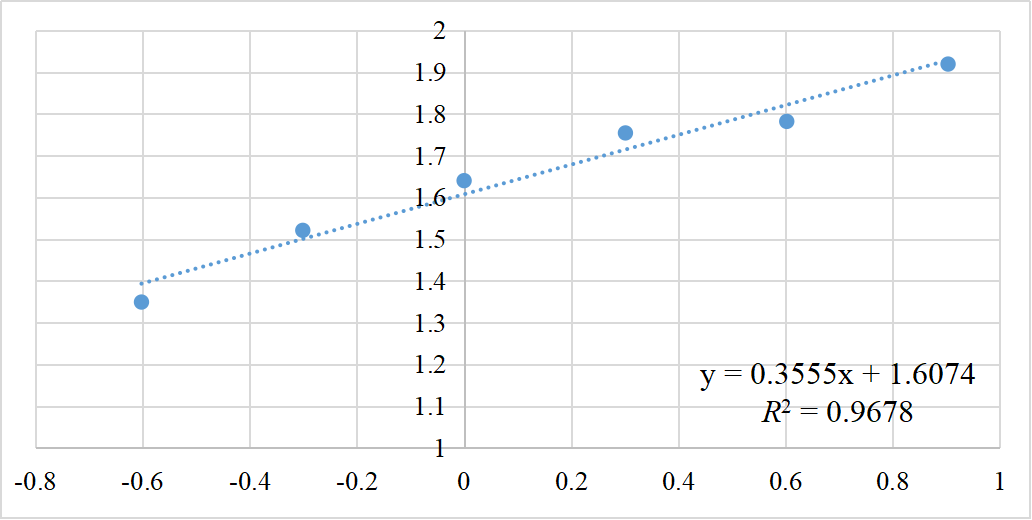


**Fig. S1** The log_10_ Cd transformed initial concentrations (mg/L) and IR in rice plants.


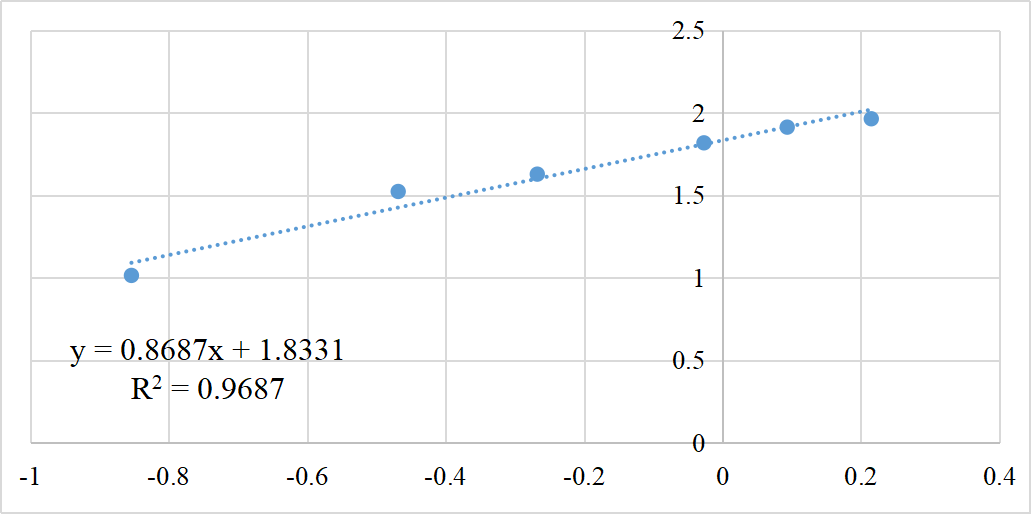


**Fig. S2** The log_10_ Na transformed initial concentrations (g/L) and IR in rice plants.

Linear regression between log_10_Cd (Log_10_Na) and IR (%) was plotted to calculate the concentrations of EC_20_, EC_50_ and EC_75_, which refer to the inhibition of 20%, 50%, and 75% of the relative growth rate in comparison to control. The calculated EC_20_, EC_50_ and EC_75_ of Cd were 0.14, 1.80, and 5.66 mg Cd/L, while the calculated EC_20_, EC_50_ and EC_75_ of Na were about 0.24, 0.70, 1.12 g Na/L. In this study, accordingly, three concentrations of Cd were designed as 0.0, 0.2, 1.8, and 5.4 mg Cd/L, while the nominal concentrations of Na were designed as 0.0, 0.2, 0.6, and 1.1 g Na/L. Apparently, in order to determine the synergistic and antagonistic effects, the effective concentrations of Cd should be in line with that of Na.

Noted that relationships between Cd or Na exposure concentrations and IR of rice could be presented by various functions. As such, the regression coefficient looks big for a linear regression do not support that such measurement is sensitivity to Cd or Na. Here, we use two methods to decide this “sensitivity”. The first is to test the correlation coefficient r with a t-test.

H_0_: ρ = 0

H_A_: ρ ≠ 0

where H_0_ indicates the “null-hypothesis”: the samples have the same mean; H_A_ indicates the the “Alternative hypothesis”: the samples have a different mean.

The underlying equation is

$$t_{test}=\frac{\left|r\right|\times\sqrt{n-2}}{\sqrt{1-r^{2}}}>t_{n-2,\alpha/2}\text{？}$$

If t_test_ > t_n-2,α/2_, then the correlation is “real”, otherwise it is by chance. The t_test_-value must be calculated, the critical t-value is found by the TINV-function of excel.

For Cd treatments (log_10_Cd) vs. IR of rice seedlings (α=0.05 or 0.01)

t-value is 8.597

T_crit_ is 2.571 (α=0.05) and 4.032 (α=0.01), respecrively.

Therefore, it is real correlation

For Na treatments (log_10_Na) vs. IR of rice seedlings (α=0.05 or 0.01)

t-value is 8.725

T_crit_ is 2.571 (α=0.05) and 4.032 (α=0.01), respecrively.

Therefore, it is real correlation

And the second method is to look up in the table of r_crit_ below. If r > r_crit_ (n-2, α), then the regression is significant.

For Cd treatments (log_10_Cd) vs. IR of rice seedlings

For n=7, df=7-2=5, α=0.05 or 0.01→r_crit(α=0.05)_=0.755 or r_crit(α=0.01)_=0.875. The calculated r is 0.9838. Therefore, the regression is significant.

For Na treatments (log_10_Na) vs. IR of rice seedlings (α=0.05 or 0.01)

For n=7, df=7-2=5, α=0.05 or 0.01→r_crit(α=0.05)_=0.755 or r_crit(α=0.01)_=0.875. The calculated r is 0.9842. Therefore, the regression is significant.

**2.7 Data analysis**

The origin Pro 8.5 was used to carried out the ANOVA and Tukey’s multiple range test as follows.

**(1) The results of ANOVA and Tukey’s multiple range test were showed in Table S3&S4 and Figure 1, respectively.**

**Table S3** The significant difference between high/medium [CO_2_] and ambient [CO_2_] under same temperature.

Table S3-1 The significant difference between high/medium [CO_2_] and ambient [CO_2_] under cold temperature with 0.0 g/L Na treatments.

|  | DF | Sum of Squares | Mean Square | F Value | Prob>F |
| --- | --- | --- | --- | --- | --- |
| Model | 2 | 25.29428 | 12.64714 | 20.69379 | 4.30166E-4 |
| Error | 9 | 5.50041 | 0.61116 |  |  |
| Total | 11 | 30.79469 |  |  |  |

At the 0.05 level, the population means are significantly different

Table S3-2 The significant difference between high/medium [CO_2_] and ambient [CO_2_] under cold temperature with 0.2 g/L Na treatments.

|  | DF | Sum of Squares | Mean Square | F Value | Prob>F |
| --- | --- | --- | --- | --- | --- |
| Model | 2 | 15.27057 | 7.63529 | 2.26259 | 0.15994 |
| Error | 9 | 30.37119 | 3.37458 |  |  |
| Total | 11 | 45.64176 |  |  |  |

At the 0.05 level, the population means are not significantly different

Table S3-3 The significant difference between high/medium [CO_2_] and ambient [CO_2_] under cold temperature with 0.0 g/L Na treatments.

|  | DF | Sum of Squares | Mean Square | F Value | Prob>F |
| --- | --- | --- | --- | --- | --- |
| Model | 2 | 37.29346 | 18.64673 | 15.12507 | 0.00132 |
| Error | 9 | 11.09552 | 1.23284 |  |  |
| Total | 11 | 48.38898 |  |  |  |

At the 0.05 level, the population means are significantly different

Table S3-4 The significant difference between high/medium [CO_2_] and ambient [CO_2_] under cold temperature with 1.1 g/L Na treatments.

|  | DF | Sum of Squares | Mean Square | F Value | Prob>F |
| --- | --- | --- | --- | --- | --- |
| Model | 2 | 26.78814 | 13.39407 | 4.4279 | 0.04582 |
| Error | 9 | 27.22432 | 3.02492 |  |  |
| Total | 11 | 54.01246 |  |  |  |

At the 0.05 level, the population means are significantly different

Table S3-5 The significant difference between high/medium [CO_2_] and ambient [CO_2_] under warm temperature with 0.0 g/L Na treatments.

|  | DF | Sum of Squares | Mean Square | F Value | Prob>F |
| --- | --- | --- | --- | --- | --- |
| Model | 2 | 336.23121 | 168.11561 | 26.34742 | 1.7297E-4 |
| Error | 9 | 57.42652 | 6.38072 |  |  |
| Total | 11 | 393.65773 |  |  |  |

At the 0.05 level, the population means are significantly different

Table S3-6 The significant difference between high/medium [CO_2_] and ambient [CO_2_] under warm temperature with 0.2 g/L Na treatments.

|  | DF | Sum of Squares | Mean Square | F Value | Prob>F |
| --- | --- | --- | --- | --- | --- |
| Model | 2 | 266.82044 | 133.41022 | 33.35695 | 6.88337E-5 |
| Error | 9 | 35.99526 | 3.99947 |  |  |
| Total | 11 | 302.8157 |  |  |  |

At the 0.05 level, the population means are significantly different

Table S3-7 The significant difference between high/medium [CO_2_] and ambient [CO_2_] under warm temperature with 0.6 g/L Na treatments.

|  | DF | Sum of Squares | Mean Square | F Value | Prob>F |
| --- | --- | --- | --- | --- | --- |
| Model | 2 | 414.31941 | 207.15971 | 155.68042 | 1.04403E-7 |
| Error | 9 | 11.97606 | 1.33067 |  |  |
| Total | 11 | 426.29547 |  |  |  |

At the 0.05 level, the population means are significantly different

Table S3-8 The significant difference between high/medium [CO_2_] and ambient [CO_2_] under warm temperature with 1.1 g/L Na treatments.

|  | DF | Sum of Squares | Mean Square | F Value | Prob>F |
| --- | --- | --- | --- | --- | --- |
| Model | 2 | 236.37249 | 118.18624 | 124.2171 | 2.79314E-7 |
| Error | 9 | 8.56304 | 0.95145 |  |  |
| Total | 11 | 244.93553 |  |  |  |

At the 0.05 level, the population means are significantly different

Table S3-9 The significant difference between high/medium [CO_2_] and ambient [CO_2_] under cold temperature with 0.0 mg/L Cd treatments.

|  | DF | Sum of Squares | Mean Square | F Value | Prob>F |
| --- | --- | --- | --- | --- | --- |
| Model | 2 | 25.29428 | 12.64714 | 20.69379 | 4.30166E-4 |
| Error | 9 | 5.50041 | 0.61116 |  |  |
| Total | 11 | 30.79469 |  |  |  |

At the 0.05 level, the population means are significantly different

Table S3-10 The significant difference between high/medium [CO_2_] and ambient [CO_2_] under cold temperature with 0.2 mg/L Cd treatments.

|  | DF | Sum of Squares | Mean Square | F Value | Prob>F |
| --- | --- | --- | --- | --- | --- |
| Model | 2 | 85.80541 | 42.9027 | 13.34717 | 0.00203 |
| Error | 9 | 28.92931 | 3.21437 |  |  |
| Total | 11 | 114.73471 |  |  |  |

At the 0.05 level, the population means are significantly different

Table S3-11 The significant difference between high/medium [CO_2_] and ambient [CO_2_] under cold temperature with 1.8 mg/L Cd treatments.

|  | DF | Sum of Squares | Mean Square | F Value | Prob>F |
| --- | --- | --- | --- | --- | --- |
| Model | 2 | 44.13748 | 22.06874 | 4.52648 | 0.04361 |
| Error | 9 | 43.8793 | 4.87548 |  |  |
| Total | 11 | 88.01678 |  |  |  |

At the 0.05 level, the population means are significantly different

Table S3-12 The significant difference between high/medium [CO_2_] and ambient [CO_2_] under cold temperature with 5.4 mg/L Cd treatments.

|  | DF | Sum of Squares | Mean Square | F Value | Prob>F |
| --- | --- | --- | --- | --- | --- |
| Model | 2 | 13.98186 | 6.99093 | 2.75077 | 0.11688 |
| Error | 9 | 22.87301 | 2.54145 |  |  |
| Total | 11 | 36.85487 |  |  |  |

At the 0.05 level, the population means are not significantly different

Table S3-13 The significant difference between high/medium [CO_2_] and ambient [CO_2_] under warm temperature with 0.0 mg/L Cd treatments.

|  | DF | Sum of Squares | Mean Square | F Value | Prob>F |
| --- | --- | --- | --- | --- | --- |
| Model | 2 | 336.23121 | 168.11561 | 26.34742 | 1.7297E-4 |
| Error | 9 | 57.42652 | 6.38072 |  |  |
| Total | 11 | 393.65773 |  |  |  |

At the 0.05 level, the population means are significantly different

Table S3-14 The significant difference between high/medium [CO_2_] and ambient [CO_2_] under warm temperature with 0.2 mg/L Cd treatments.

|  | DF | Sum of Squares | Mean Square | F Value | Prob>F |
| --- | --- | --- | --- | --- | --- |
| Model | 2 | 223.75756 | 111.87878 | 99.58604 | 7.26424E-7 |
| Error | 9 | 10.11095 | 1.12344 |  |  |
| Total | 11 | 233.86851 |  |  |  |

At the 0.05 level, the population means are significantly different

Table S3-15 The significant difference between high/medium [CO_2_] and ambient [CO_2_] under warm temperature with 1.8 mg/L Cd treatments.

|  | DF | Sum of Squares | Mean Square | F Value | Prob>F |
| --- | --- | --- | --- | --- | --- |
| Model | 2 | 382.64258 | 191.32129 | 87.40203 | 1.27202E-6 |
| Error | 9 | 19.70082 | 2.18898 |  |  |
| Total | 11 | 402.3434 |  |  |  |

At the 0.05 level, the population means are significantly different

Table S3-16 The significant difference between high/medium [CO_2_] and ambient [CO_2_] under warm temperature with 5.4 mg/L Cd treatments.

|  | DF | Sum of Squares | Mean Square | F Value | Prob>F |
| --- | --- | --- | --- | --- | --- |
| Model | 2 | 460.14746 | 230.07373 | 90.2767 | 1.10739E-6 |
| Error | 9 | 22.93686 | 2.54854 |  |  |
| Total | 11 | 483.08432 |  |  |  |

At the 0.05 level, the population means are significantly different

Table S3-17 The significant difference between high/medium [CO_2_] and ambient [CO_2_] under cold temperature with 0.0 g Na/L+0.0 mg Cd/L treatments.

|  | DF | Sum of Squares | Mean Square | F Value | Prob>F |
| --- | --- | --- | --- | --- | --- |
| Model | 2 | 25.29428 | 12.64714 | 20.69379 | 4.30166E-4 |
| Error | 9 | 5.50041 | 0.61116 |  |  |
| Total | 11 | 30.79469 |  |  |  |

At the 0.05 level, the population means are significantly different

Table S3-18 The significant difference between high/medium [CO_2_] and ambient [CO_2_] under cold temperature with 0.2 g Na/L+0.2 mg Cd/L treatments.

|  | DF | Sum of Squares | Mean Square | F Value | Prob>F |
| --- | --- | --- | --- | --- | --- |
| Model | 2 | 93.29765 | 46.64883 | 21.45997 | 3.75913E-4 |
| Error | 9 | 19.56384 | 2.17376 |  |  |
| Total | 11 | 112.8615 |  |  |  |

At the 0.05 level, the population means are significantly different

Table S3-19 The significant difference between high/medium [CO_2_] and ambient [CO_2_] under cold temperature with 0.6 g Na/L+1.8 mg Cd/L treatments.

|  | DF | Sum of Squares | Mean Square | F Value | Prob>F |
| --- | --- | --- | --- | --- | --- |
| Model | 2 | 16.0614 | 8.0307 | 3.54488 | 0.07322 |
| Error | 9 | 20.38891 | 2.26543 |  |  |
| Total | 11 | 36.45031 |  |  |  |

At the 0.05 level, the population means are not significantly different

Table S3-20 The significant difference between high/medium [CO_2_] and ambient [CO_2_] under cold temperature with 1.1 g Na/L+5.4 mg Cd/L treatments.

|  | DF | Sum of Squares | Mean Square | F Value | Prob>F |
| --- | --- | --- | --- | --- | --- |
| Model | 2 | 4.88407 | 2.44203 | 1.91688 | 0.20253 |
| Error | 9 | 11.46567 | 1.27396 |  |  |
| Total | 11 | 16.34974 |  |  |  |

At the 0.05 level, the population means are not significantly different

Table S3-21 The significant difference between high/medium [CO_2_] and ambient [CO_2_] under warm temperature with 0.0 g Na/L+0.0 mg Cd/L treatments.

|  | DF | Sum of Squares | Mean Square | F Value | Prob>F |
| --- | --- | --- | --- | --- | --- |
| Model | 2 | 336.23121 | 168.11561 | 26.34742 | 1.7297E-4 |
| Error | 9 | 57.42652 | 6.38072 |  |  |
| Total | 11 | 393.65773 |  |  |  |

At the 0.05 level, the population means are significantly different

Table S3-21 The significant difference between high/medium [CO_2_] and ambient [CO_2_] under warm temperature with 0.2 g Na/L+0.2 mg Cd/L treatments.

|  | DF | Sum of Squares | Mean Square | F Value | Prob>F |
| --- | --- | --- | --- | --- | --- |
| Model | 2 | 288.72662 | 144.36331 | 27.09782 | 1.55238E-4 |
| Error | 9 | 47.94739 | 5.32749 |  |  |
| Total | 11 | 336.67401 |  |  |  |

At the 0.05 level, the population means are significantly different

Table S3-22 The significant difference between high/medium [CO_2_] and ambient [CO_2_] under warm temperature with 0.6 g Na/L+1.8 mg Cd/L treatments.

|  | DF | Sum of Squares | Mean Square | F Value | Prob>F |
| --- | --- | --- | --- | --- | --- |
| Model | 2 | 128.51711 | 64.25856 | 76.13745 | 2.29108E-6 |
| Error | 9 | 7.59583 | 0.84398 |  |  |
| Total | 11 | 136.11294 |  |  |  |

At the 0.05 level, the population means are significantly different

**Table S3-24 The significant difference between high/medium [CO_2_] and ambient [CO_2_] under warm temperature with 1.1 g Na/L+5.4 mg Cd/L treatments.**

|  | DF | Sum of Squares | Mean Square | F Value | Prob>F |
| --- | --- | --- | --- | --- | --- |
| Model | 2 | 149.17165 | 74.58583 | 21.87544 | 3.49991E-4 |
| Error | 9 | 30.68612 | 3.40957 |  |  |
| Total | 11 | 179.85777 |  |  |  |

At the 0.05 level, the population means are significantly different

**Table S4** The significant difference between Cd, Na, Cd+Na treatments and control.

Table S4-1 The significant difference between Na treatments (0.2, 0.6, and 1.1 g/L) and 0.0 g/L treatment under cold temperature and ambient [CO_2_].

|  | DF | Sum of Squares | Mean Square | F Value | Prob>F |
| --- | --- | --- | --- | --- | --- |
| Model | 3 | 513.59247 | 171.19749 | 192.42204 | 2.07324E-10 |
| Error | 12 | 10.67638 | 0.8897 |  |  |
| Total | 15 | 524.26884 |  |  |  |

At the 0.05 level, the population means are significantly different

Table S4-2 The significant difference between Na treatments (0.2, 0.6, and 1.1 g/L) and 0.0 g/L treatment under cold temperature and medium [CO_2_].

|  | DF | Sum of Squares | Mean Square | F Value | Prob>F |
| --- | --- | --- | --- | --- | --- |
| Model | 3 | 612.31954 | 204.10651 | 68.60816 | 8.00196E-8 |
| Error | 12 | 35.69952 | 2.97496 |  |  |
| Total | 15 | 648.01906 |  |  |  |

At the 0.05 level, the population means are significantly different

Table S4-3 The significant difference between Na treatments (0.2, 0.6, and 1.1 g/L) and 0.0 g/L treatment under cold temperature and high [CO_2_].

|  | DF | Sum of Squares | Mean Square | F Value | Prob>F |
| --- | --- | --- | --- | --- | --- |
| Model | 3 | 490.40547 | 163.46849 | 70.52251 | 6.84952E-8 |
| Error | 12 | 27.81554 | 2.31796 |  |  |
| Total | 15 | 518.22101 |  |  |  |

At the 0.05 level, the population means are significantly different

Table S4-4 The significant difference between Na treatments (0.2, 0.6, and 1.1 g/L) and 0.0 g/L treatment under warm temperature and ambient [CO_2_].

|  | DF | Sum of Squares | Mean Square | F Value | Prob>F |
| --- | --- | --- | --- | --- | --- |
| Model | 3 | 629.9589 | 209.9863 | 138.83915 | 1.39719E-9 |
| Error | 12 | 18.14932 | 1.51244 |  |  |
| Total | 15 | 648.10821 |  |  |  |

At the 0.05 level, the population means are significantly different

Table S4-5 The significant difference between Na treatments (0.2, 0.6, and 1.1 g/L) and 0.0 g/L treatment under warm temperature and medium [CO_2_].

|  | DF | Sum of Squares | Mean Square | F Value | Prob>F |
| --- | --- | --- | --- | --- | --- |
| Model | 3 | 891.95628 | 297.31876 | 140.76683 | 1.28942E-9 |
| Error | 12 | 25.34564 | 2.11214 |  |  |
| Total | 15 | 917.30192 |  |  |  |

At the 0.05 level, the population means are significantly different

Table S4-6 The significant difference between Na treatments (0.2, 0.6, and 1.1 g/L) and 0.0 g/L treatment under warm temperature and high [CO_2_].

|  | DF | Sum of Squares | Mean Square | F Value | Prob>F |
| --- | --- | --- | --- | --- | --- |
| Model | 3 | 746.19771 | 248.73257 | 42.35793 | 1.16461E-6 |
| Error | 12 | 70.46592 | 5.87216 |  |  |
| Total | 15 | 816.66363 |  |  |  |

At the 0.05 level, the population means are significantly different

Table S4-7 The significant difference between Cd treatments (0.2, 1.8, and 5.4 mg/L) and 0.0 mg/L treatment under cold temperature and ambient [CO_2_].

|  | DF | Sum of Squares | Mean Square | F Value | Prob>F |
| --- | --- | --- | --- | --- | --- |
| Model | 3 | 536.66633 | 178.88878 | 124.52919 | 2.62865E-9 |
| Error | 12 | 17.23825 | 1.43652 |  |  |
| Total | 15 | 553.90458 |  |  |  |

At the 0.05 level, the population means are significantly different

Table S4-8 The significant difference between Cd treatments (0.2, 1.8, and 5.4 mg/L) and 0.0 mg/L treatment under cold temperature and medium [CO_2_].

|  | DF | Sum of Squares | Mean Square | F Value | Prob>F |
| --- | --- | --- | --- | --- | --- |
| Model | 3 | 573.09686 | 191.03229 | 58.86273 | 1.89266E-7 |
| Error | 12 | 38.94463 | 3.24539 |  |  |
| Total | 15 | 612.04149 |  |  |  |

At the 0.05 level, the population means are significantly different

Table S4-9 The significant difference between Cd treatments (0.2, 1.8, and 5.4 mg/L) and 0.0 mg/L treatment under cold temperature and high [CO_2_].

|  | DF | Sum of Squares | Mean Square | F Value | Prob>F |
| --- | --- | --- | --- | --- | --- |
| Model | 3 | 626.64907 | 208.88302 | 55.7032 | 2.57503E-7 |
| Error | 12 | 44.99914 | 3.74993 |  |  |
| Total | 15 | 671.64821 |  |  |  |

At the 0.05 level, the population means are significantly different

Table S4-10 The significant difference between Cd treatments (0.2, 1.8, and 5.4 mg/L) and 0.0 mg/L treatment under warm temperature and ambient [CO_2_].

|  | DF | Sum of Squares | Mean Square | F Value | Prob>F |
| --- | --- | --- | --- | --- | --- |
| Model | 3 | 723.92456 | 241.30819 | 206.5875 | 1.36601E-10 |
| Error | 12 | 14.01681 | 1.16807 |  |  |
| Total | 15 | 737.94137 |  |  |  |

At the 0.05 level, the population means are significantly different

Table S4-11 The significant difference between Cd treatments (0.2, 1.8, and 5.4 mg/L) and 0.0 mg/L treatment under warm temperature and medium [CO_2_].

|  | DF | Sum of Squares | Mean Square | F Value | Prob>F |
| --- | --- | --- | --- | --- | --- |
| Model | 3 | 920.39346 | 306.79782 | 143.02293 | 1.17539E-9 |
| Error | 12 | 25.74114 | 2.1451 |  |  |
| Total | 15 | 946.13461 |  |  |  |

At the 0.05 level, the population means are significantly different

Table S4-12 The significant difference between Cd treatments (0.2, 1.8, and 5.4 mg/L) and 0.0 mg/L treatment under warm temperature and high [CO_2_].

|  | DF | Sum of Squares | Mean Square | F Value | Prob>F |
| --- | --- | --- | --- | --- | --- |
| Model | 3 | 545.70478 | 181.90159 | 30.99839 | 6.20763E-6 |
| Error | 12 | 70.41719 | 5.8681 |  |  |
| Total | 15 | 616.12197 |  |  |  |

At the 0.05 level, the population means are significantly different

Table S4-13 The significant difference between Cd+Na treatments (0.2 mg Cd/L+0.2 g Na/L, 1.8 mg Cd/L+0.6 g Na/L, and 5.4 mg Cd/L+1.1 g Na/L) and 0.0 mg Cd/L+0.0 g Na/L treatment under cold temperature and ambient [CO_2_].

|  | DF | Sum of Squares | Mean Square | F Value | Prob>F |
| --- | --- | --- | --- | --- | --- |
| Model | 3 | 499.32408 | 166.44136 | 121.13563 | 3.08529E-9 |
| Error | 12 | 16.4881 | 1.37401 |  |  |
| Total | 15 | 515.81218 |  |  |  |

At the 0.05 level, the population means are significantly different

Table S4-14 The significant difference between Cd+Na treatments (0.2 mg Cd/L+0.2 g Na/L, 1.8 mg Cd/L+0.6 g Na/L, and 5.4 mg Cd/L+1.1 g Na/L) and 0.0 mg Cd/L+0.0 g Na/L treatment under cold temperature and medium [CO_2_].

|  | DF | Sum of Squares | Mean Square | F Value | Prob>F |
| --- | --- | --- | --- | --- | --- |
| Model | 3 | 615.30475 | 205.10158 | 77.55775 | 3.99481E-8 |
| Error | 12 | 31.73402 | 2.6445 |  |  |
| Total | 15 | 647.03876 |  |  |  |

At the 0.05 level, the population means are significantly different

Table S4-15 The significant difference between Cd+Na treatments (0.2 mg Cd/L+0.2 g Na/L, 1.8 mg Cd/L+0.6 g Na/L, and 5.4 mg Cd/L+1.1 g Na/L) and 0.0 mg Cd/L+0.0 g Na/L treatment under cold temperature and high [CO_2_].

|  | DF | Sum of Squares | Mean Square | F Value | Prob>F |
| --- | --- | --- | --- | --- | --- |
| Model | 3 | 691.7296 | 230.57653 | 318.15674 | 1.06878E-11 |
| Error | 12 | 8.69671 | 0.72473 |  |  |
| Total | 15 | 700.42632 |  |  |  |

At the 0.05 level, the population means are significantly different

Table S4-16 The significant difference between Cd+Na treatments (0.2 mg Cd/L+0.2 g Na/L, 1.8 mg Cd/L+0.6 g Na/L, and 5.4 mg Cd/L+1.1 g Na/L) and 0.0 mg Cd/L+0.0 g Na/L treatment under warm temperature and ambient [CO_2_].

|  | DF | Sum of Squares | Mean Square | F Value | Prob>F |
| --- | --- | --- | --- | --- | --- |
| Model | 3 | 669.30205 | 223.10068 | 197.96349 | 1.75493E-10 |
| Error | 12 | 13.52375 | 1.12698 |  |  |
| Total | 15 | 682.8258 |  |  |  |

At the 0.05 level, the population means are significantly different

Table S4-17 The significant difference between Cd+Na treatments (0.2 mg Cd/L+0.2 g Na/L, 1.8 mg Cd/L+0.6 g Na/L, and 5.4 mg Cd/L+1.1 g Na/L) and 0.0 mg Cd/L+0.0 g Na/L treatment under warm temperature and medium [CO_2_].

|  | DF | Sum of Squares | Mean Square | F Value | Prob>F |
| --- | --- | --- | --- | --- | --- |
| Model | 3 | 1017.20471 | 339.06824 | 128.15838 | 2.22504E-9 |
| Error | 12 | 31.74836 | 2.6457 |  |  |
| Total | 15 | 1048.95307 |  |  |  |

At the 0.05 level, the population means are significantly different

Table S4-18 The significant difference between Cd+Na treatments (0.2 mg Cd/L+0.2 g Na/L, 1.8 mg Cd/L+0.6 g Na/L, and 5.4 mg Cd/L+1.1 g Na/L) and 0.0 mg Cd/L+0.0 g Na/L treatment under warm temperature and high [CO_2_].

|  | DF | Sum of Squares | Mean Square | F Value | Prob>F |
| --- | --- | --- | --- | --- | --- |
| Model | 3 | 1142.68486 | 380.89495 | 46.45828 | 7.02643E-7 |
| Error | 12 | 98.38374 | 8.19865 |  |  |
| Total | 15 | 1241.0686 |  |  |  |

At the 0.05 level, the population means are significantly different

**(2)** **The results of ANOVA and Tukey’s multiple range test were showed in Table S5&S6 and Figure 2, respectively.**

**Table S5** The significant difference between high/medium [CO_2_] and ambient [CO_2_] under same temperature.

Table S5-1 The significant difference between high/medium [CO_2_] and ambient [CO_2_] under cold temperature with 0.2 mg/L Cd treatment in rice roots.

|  | DF | Sum of Squares | Mean Square | F Value | Prob>F |
| --- | --- | --- | --- | --- | --- |
| Model | 2 | 1.65391 | 0.82695 | 0.01529 | 2 |
| Error | 9 | 486.69186 | 54.07687 |  | 9 |
| Total | 11 | 488.34577 |  |  | 11 |

At the 0.05 level, the population means are not significantly different

Table S5-2 The significant difference between high/medium [CO_2_] and ambient [CO_2_] under cold temperature with 1.8 mg/L Cd treatment in rice roots.

|  | DF | Sum of Squares | Mean Square | F Value | Prob>F |
| --- | --- | --- | --- | --- | --- |
| Model | 2 | 268117.0954 | 134058.5477 | 12.41272 | 2 |
| Error | 9 | 97200.81927 | 10800.09103 |  | 9 |
| Total | 11 | 365317.91467 |  |  | 11 |

At the 0.05 level, the population means are significantly different

Table S5-3 The significant difference between high/medium [CO_2_] and ambient [CO_2_] under cold temperature with 5.4 mg/L Cd treatment in rice roots.

|  | DF | Sum of Squares | Mean Square | F Value | Prob>F |
| --- | --- | --- | --- | --- | --- |
| Model | 2 | 1.85921E6 | 929606.52797 | 9.40528 | 2 |
| Error | 9 | 889549.4985 | 98838.83317 |  | 9 |
| Total | 11 | 2.74876E6 |  |  | 11 |

At the 0.05 level, the population means are significantly different

Table S5-4 The significant difference between high/medium [CO_2_] and ambient [CO_2_] under warm temperature with 0.2 mg/L Cd treatment in rice roots.

|  | DF | Sum of Squares | Mean Square | F Value | Prob>F |
| --- | --- | --- | --- | --- | --- |
| Model | 2 | 1804.5346 | 902.2673 | 6.6495 | 0.01686 |
| Error | 9 | 1221.20514 | 135.68946 |  |  |
| Total | 11 | 3025.73973 |  |  |  |

At the 0.05 level, the population means are significantly different

Table S5-5 The significant difference between high/medium [CO_2_] and ambient [CO_2_] under warm temperature with 1.8 mg/L Cd treatment in rice roots.

|  | DF | Sum of Squares | Mean Square | F Value | Prob>F |
| --- | --- | --- | --- | --- | --- |
| Model | 2 | 210519.07416 | 105259.53708 | 3.84944 | 0.06194 |
| Error | 9 | 246096.78924 | 27344.08769 |  |  |
| Total | 11 | 456615.8634 |  |  |  |

At the 0.05 level, the population means are not significantly different

Table S5-6 The significant difference between high/medium [CO_2_] and ambient [CO_2_] under warm temperature with 5.4 mg/L Cd treatment in rice roots.

|  | DF | Sum of Squares | Mean Square | F Value | Prob>F |
| --- | --- | --- | --- | --- | --- |
| Model | 2 | 1.84277E6 | 921386.85109 | 11.6152 | 0.00321 |
| Error | 9 | 713933.90635 | 79325.98959 |  |  |
| Total | 11 | 2.55671E6 |  |  |  |

At the 0.05 level, the population means are significantly different

Table S5-7 The significant difference between high/medium [CO_2_] and ambient [CO_2_] under cold temperature with 0.2 mg/L Cd treatment in rice shoots.

|  | DF | Sum of Squares | Mean Square | F Value | Prob>F |
| --- | --- | --- | --- | --- | --- |
| Model | 2 | 12.94918 | 6.47459 | 2.1022 | 0.17818 |
| Error | 9 | 27.71925 | 3.07992 |  |  |
| Total | 11 | 40.66843 |  |  |  |

At the 0.05 level, the population means are not significantly different

Table S5-8 The significant difference between high/medium [CO_2_] and ambient [CO_2_] under cold temperature with 1.8 mg/L Cd treatment in rice shoots.

|  | DF | Sum of Squares | Mean Square | F Value | Prob>F |
| --- | --- | --- | --- | --- | --- |
| Model | 2 | 34.49434 | 17.24717 | 4.2753 | 0.04952 |
| Error | 9 | 36.30727 | 4.03414 |  |  |
| Total | 11 | 70.8016 |  |  |  |

At the 0.05 level, the population means are significantly different

Table S5-9 The significant difference between high/medium [CO_2_] and ambient [CO_2_] under cold temperature with 5.4 mg/L Cd treatment in rice shoots.

|  | DF | Sum of Squares | Mean Square | F Value | Prob>F |
| --- | --- | --- | --- | --- | --- |
| Model | 2 | 430.84529 | 215.42265 | 36.98928 | 2 |
| Error | 9 | 52.41529 | 5.82392 |  | 9 |
| Total | 11 | 483.26058 |  |  | 11 |

At the 0.05 level, the population means are significantly different

Table S5-10 The significant difference between high/medium [CO_2_] and ambient [CO_2_] under warm temperature with 0.2 mg/L Cd treatment in rice shoots.

|  | DF | Sum of Squares | Mean Square | F Value | Prob>F |
| --- | --- | --- | --- | --- | --- |
| Model | 2 | 7.63789E6 | 3.81894E6 | 634.40263 | 2.06538E-10 |
| Error | 9 | 54177.72808 | 6019.74756 |  |  |
| Total | 11 | 7.69207E6 |  |  |  |

At the 0.05 level, the population means are significantly different

Table S5-11 The significant difference between high/medium [CO_2_] and ambient [CO_2_] under warm temperature with 1.8 mg/L Cd treatment in rice shoots.

|  | DF | Sum of Squares | Mean Square | F Value | Prob>F |
| --- | --- | --- | --- | --- | --- |
| Model | 2 | 31.41832 | 15.70916 | 2.68877 | 0.12148 |
| Error | 9 | 52.58262 | 5.84251 |  |  |
| Total | 11 | 84.00094 |  |  |  |

At the 0.05 level, the population means are not significantly different

Table S5-12 The significant difference between high/medium [CO_2_] and ambient [CO_2_] under warm temperature with 5.4 mg/L Cd treatment in rice shoots.

|  | DF | Sum of Squares | Mean Square | F Value | Prob>F |
| --- | --- | --- | --- | --- | --- |
| Model | 2 | 13.60759 | 6.8038 | 0.81289 | 0.47366 |
| Error | 9 | 75.32867 | 8.36985 |  |  |
| Total | 11 | 88.93626 |  |  |  |

At the 0.05 level, the population means are not significantly different

Table S5-13 The significant difference between high/medium [CO_2_] and ambient [CO_2_] under cold temperature with 0.2 g Na/L+0.2 mg Cd/L treatment in rice roots.

|  | DF | Sum of Squares | Mean Square | F Value | Prob>F |
| --- | --- | --- | --- | --- | --- |
| Model | 2 | 977.16255 | 488.58127 | 1.82113 | 0.21671 |
| Error | 9 | 2414.55568 | 268.28396 |  |  |
| Total | 11 | 3391.71823 |  |  |  |

At the 0.05 level, the population means are not significantly different

Table S5-14 The significant difference between high/medium [CO_2_] and ambient [CO_2_] under cold temperature with 0.6 g Na/L+1.8 mg Cd/L treatment in rice roots.

|  | DF | Sum of Squares | Mean Square | F Value | Prob>F |
| --- | --- | --- | --- | --- | --- |
| Model | 2 | 105565.36212 | 52782.68106 | 11.01396 | 0.00381 |
| Error | 9 | 43131.08347 | 4792.34261 |  |  |
| Total | 11 | 148696.44559 |  |  |  |

At the 0.05 level, the population means are significantly different

Table S5-15 The significant difference between high/medium [CO_2_] and ambient [CO_2_] under cold temperature with 1.1 g Na/L+5.4 mg Cd/L treatment in rice roots.

|  | DF | Sum of Squares | Mean Square | F Value | Prob>F |
| --- | --- | --- | --- | --- | --- |
| Model | 2 | 5.54738E6 | 2.77369E6 | 31.84774 | 8.26623E-5 |
| Error | 9 | 783830.15212 | 87092.23912 |  |  |
| Total | 11 | 6.33121E6 |  |  |  |

At the 0.05 level, the population means are significantly different

Table S5-16 The significant difference between high/medium [CO_2_] and ambient [CO_2_] under warm temperature with 0.2 g Na/L+0.2 mg Cd/L treatment in rice roots.

|  | DF | Sum of Squares | Mean Square | F Value | Prob>F |
| --- | --- | --- | --- | --- | --- |
| Model | 2 | 821.56041 | 410.78021 | 0.98631 | 0.40992 |
| Error | 9 | 3748.35482 | 416.48387 |  |  |
| Total | 11 | 4569.91523 |  |  |  |

At the 0.05 level, the population means are not significantly different

Table S5-17 The significant difference between high/medium [CO_2_] and ambient [CO_2_] under warm temperature with 0.6 g Na/L+1.8 mg Cd/L treatment in rice roots.

|  | DF | Sum of Squares | Mean Square | F Value | Prob>F |
| --- | --- | --- | --- | --- | --- |
| Model | 2 | 119683.89819 | 59841.9491 | 2.03683 | 0.18634 |
| Error | 9 | 264419.37159 | 29379.93018 |  |  |
| Total | 11 | 384103.26978 |  |  |  |

At the 0.05 level, the population means are not significantly different

Table S5-18 The significant difference between high/medium [CO_2_] and ambient [CO_2_] under warm temperature with 1.1 g Na/L+5.4 mg Cd/L treatment in rice roots.

|  | DF | Sum of Squares | Mean Square | F Value | Prob>F |
| --- | --- | --- | --- | --- | --- |
| Model | 2 | 1.67138E6 | 835687.52857 | 10.98248 | 0.00385 |
| Error | 9 | 684835.37338 | 76092.81926 |  |  |
| Total | 11 | 2.35621E6 |  |  |  |

At the 0.05 level, the population means are significantly different

Table S5-19 The significant difference between high/medium [CO_2_] and ambient [CO_2_] under cold temperature with 0.2 g Na/L+0.2 mg Cd/L treatment in rice shoots.

|  | DF | Sum of Squares | Mean Square | F Value | Prob>F |
| --- | --- | --- | --- | --- | --- |
| Model | 2 | 41.3701 | 20.68505 | 9.82143 | 0.00546 |
| Error | 9 | 18.95502 | 2.10611 |  |  |
| Total | 11 | 60.32512 |  |  |  |

At the 0.05 level, the population means are significantly different

Table S5-20 The significant difference between high/medium [CO_2_] and ambient [CO_2_] under cold temperature with 0.6 g Na/L+1.8 mg Cd/L treatment in rice shoots.

|  | DF | Sum of Squares | Mean Square | F Value | Prob>F |
| --- | --- | --- | --- | --- | --- |
| Model | 2 | 61.18464 | 30.59232 | 4.14325 | 0.05302 |
| Error | 9 | 66.4528 | 7.38364 |  |  |
| Total | 11 | 127.63744 |  |  |  |

At the 0.05 level, the population means are not significantly different

Table S5-21 The significant difference between high/medium [CO_2_] and ambient [CO_2_] under cold temperature with 1.1 g Na/L+5.4 mg Cd/L treatment in rice shoots.

|  | DF | Sum of Squares | Mean Square | F Value | Prob>F |
| --- | --- | --- | --- | --- | --- |
| Model | 2 | 155.70473 | 77.85236 | 2.44097 | 0.14225 |
| Error | 9 | 287.04592 | 31.89399 |  |  |
| Total | 11 | 442.75065 |  |  |  |

At the 0.05 level, the population means are not significantly different

Table S5-22 The significant difference between high/medium [CO_2_] and ambient [CO_2_] under warm temperature with 0.2 g Na/L+0.2 mg Cd/L treatment in rice shoots.

|  | DF | Sum of Squares | Mean Square | F Value | Prob>F |
| --- | --- | --- | --- | --- | --- |
| Model | 2 | 26.99118 | 13.49559 | 9.83704 | 0.00544 |
| Error | 9 | 12.34724 | 1.37192 |  |  |
| Total | 11 | 39.33842 |  |  |  |

At the 0.05 level, the population means are significantly different

Table S5-23 The significant difference between high/medium [CO_2_] and ambient [CO_2_] under warm temperature with 0.6 g Na/L+1.8 mg Cd/L treatment in rice shoots.

|  | DF | Sum of Squares | Mean Square | F Value | Prob>F |
| --- | --- | --- | --- | --- | --- |
| Model | 2 | 2.43002 | 1.21501 | 0.39222 | 0.68656 |
| Error | 9 | 27.88028 | 3.09781 |  |  |
| Total | 11 | 30.31029 |  |  |  |

At the 0.05 level, the population means are not significantly different

Table S5-24 The significant difference between high/medium [CO_2_] and ambient [CO_2_] under warm temperature with 1.1 g Na/L+5.4 mg Cd/L treatment in rice shoots.

|  | DF | Sum of Squares | Mean Square | F Value | Prob>F |
| --- | --- | --- | --- | --- | --- |
| Model | 2 | 5.99237 | 2.99618 | 0.3855 | 0.69082 |
| Error | 9 | 69.95017 | 7.77224 |  |  |
| Total | 11 | 75.94254 |  |  |  |

At the 0.05 level, the population means are not significantly different

**Table S6** The significant difference between Cd treatments and Cd+Na treatments under same temperature and [CO_2_] conditions.

Table S6-1 The significant difference between Cd treatments and Cd+Na treatments under cold temperature and ambient [CO_2_] conditions in rice roots.

|  | DF | Sum of Squares | Mean Square | F Value | Prob>F |
| --- | --- | --- | --- | --- | --- |
| (1) 0.2 mg Cd/L vs. 0.2 mg Cd Cd/L+0.2 g Na/L | | | | | |
| Model | 1 | 296.78281 | 296.78281 | 3.47534 | 0.11157 |
| Error | 6 | 512.38131 | 85.39689 |  |  |
| Total | 7 | 809.16412 |  |  |  |
| (2) 1.8 mg Cd/L vs. 1.8 mg Cd Cd/L+0.6 g Na/L | | | | | |
| Model | 1 | 21759.5265 | 21759.5265 | 25.75263 | 0.00228 |
| Error | 6 | 5069.66243 | 844.94374 |  |  |
| Total | 7 | 26829.18893 |  |  |  |
| (3) 5.4 mg Cd/L vs. 5.4 mg Cd Cd/L+1.1 g Na/L | | | | | |
| Model | 1 | 282598.94961 | 282598.94961 | 14.51856 | 0.00886 |
| Error | 6 | 116787.9982 | 19464.66637 |  |  |
| Total | 7 | 399386.94781 |  |  |  |

(1) At the 0.05 level, the population means are not significantly different

(2) At the 0.05 level, the population means are significantly different

(3) At the 0.05 level, the population means are significantly different

Table S6-2 The significant difference between Cd treatments and Cd+Na treatments under cold temperature and medium [CO_2_] conditions in rice roots.

|  | DF | Sum of Squares | Mean Square | F Value | Prob>F |
| --- | --- | --- | --- | --- | --- |
| (1) 0.2 mg Cd/L vs. 0.2 mg Cd Cd/L+0.2 g Na/L | | | | | |
| Model | 1 | 2053.72719 | 2053.72719 | 7.07797 | 0.0375 |
| Error | 6 | 1740.94474 | 290.15746 |  |  |
| Total | 7 | 3794.67194 |  |  |  |
| (2) 1.8 mg Cd/L vs. 1.8 mg Cd Cd/L+0.6 g Na/L | | | | | |
| Model | 1 | 112726.50126 | 112726.50126 | 14.21739 | 0.00928 |
| Error | 6 | 47572.65347 | 7928.77558 |  |  |
| Total | 7 | 160299.15473 |  |  |  |
| (3) 5.4 mg Cd/L vs. 5.4 mg Cd Cd/L+1.1 g Na/L | | | | | |
| Model | 1 | 151569.5448 | 151569.5448 | 0.88081 | 0.3842 |
| Error | 6 | 1.03247E6 | 172078.90701 |  |  |
| Total | 7 | 1.18404E6 |  |  |  |

(1) At the 0.05 level, the population means are significantly different

(2) At the 0.05 level, the population means are significantly different

(3) At the 0.05 level, the population means are not significantly different

Table S6-3 The significant difference between Cd treatments and Cd+Na treatments under cold temperature and high [CO_2_] conditions in rice roots.

|  | DF | Sum of Squares | Mean Square | F Value | Prob>F |
| --- | --- | --- | --- | --- | --- |
| (1) 0.2 mg Cd/L vs. 0.2 mg Cd Cd/L+0.2 g Na/L | | | | | |
| Model | 1 | 1999.90639 | 1999.90639 | 18.5199 | 0.00507 |
| Error | 6 | 647.92149 | 107.98691 |  |  |
| Total | 7 | 2647.82787 |  |  |  |
| (2) 1.8 mg Cd/L vs. 1.8 mg Cd Cd/L+0.6 g Na/L | | | | | |
| Model | 1 | 39776.69003 | 39776.69003 | 2.72165 | 0.15009 |
| Error | 6 | 87689.58684 | 14614.93114 |  |  |
| Total | 7 | 127466.27687 |  |  |  |
| (3) 5.4 mg Cd/L vs. 5.4 mg Cd Cd/L+1.1 g Na/L | | | | | |
| Model | 1 | 154758.23079 | 154758.23079 | 1.77164 | 0.23152 |
| Error | 6 | 524118.21038 | 87353.03506 |  |  |
| Total | 7 | 678876.44117 |  |  |  |

(1) At the 0.05 level, the population means are significantly different

(2) At the 0.05 level, the population means are not significantly different

(3) At the 0.05 level, the population means are not significantly different

Table S6-4 The significant difference between Cd treatments and Cd+Na treatments under warm temperature and ambient [CO_2_] conditions in rice roots.

|  | DF | Sum of Squares | Mean Square | F Value | Prob>F |
| --- | --- | --- | --- | --- | --- |
| (1) 0.2 mg Cd/L vs. 0.2 mg Cd Cd/L+0.2 g Na/L | | | | | |
| Model | 1 | 501.59801 | 501.59801 | 3.57347 | 0.1076 |
| Error | 6 | 842.20402 | 140.36734 |  |  |
| Total | 7 | 1343.80204 |  |  |  |
| (2) 1.8 mg Cd/L vs. 1.8 mg Cd Cd/L+0.6 g Na/L | | | | | |
| Model | 1 | 138686.44192 | 138686.44192 | 11.43089 | 0.01484 |
| Error | 6 | 72795.63487 | 12132.60581 |  |  |
| Total | 7 | 211482.07679 |  |  |  |
| (3) 5.4 mg Cd/L vs. 5.4 mg Cd Cd/L+1.1 g Na/L | | | | | |
| Model | 1 | 1.25872E6 | 1.25872E6 | 11.4559 | 0.01477 |
| Error | 6 | 659252.8477 | 109875.47462 |  |  |
| Total | 7 | 1.91798E6 |  |  |  |

(1) At the 0.05 level, the population means are not significantly different

(2) At the 0.05 level, the population means are not significantly different

(3) At the 0.05 level, the population means are significantly different

Table S6-5 The significant difference between Cd treatments and Cd+Na treatments under warm temperature and medium [CO_2_] conditions in rice roots.

|  | DF | Sum of Squares | Mean Square | F Value | Prob>F |
| --- | --- | --- | --- | --- | --- |
| (1) 0.2 mg Cd/L vs. 0.2 mg Cd Cd/L+0.2 g Na/L | | | | | |
| Model | 1 | 24.17466 | 24.17466 | 0.08313 | 0.7828 |
| Error | 6 | 1744.82382 | 290.80397 |  |  |
| Total | 7 | 1768.99848 |  |  |  |
| (2) 1.8 mg Cd/L vs. 1.8 mg Cd Cd/L+0.6 g Na/L | | | | | |
| Model | 1 | 136555.26404 | 136555.26404 | 3.06988 | 0.13031 |
| Error | 6 | 266893.3284 | 44482.2214 |  |  |
| Total | 7 | 403448.59244 |  |  |  |
| (3) 5.4 mg Cd/L vs. 5.4 mg Cd Cd/L+1.1 g Na/L | | | | | |
| Model | 1 | 1.29279E6 | 1.29279E6 | 17.47046 | 0.00581 |
| Error | 6 | 443991.28386 | 73998.54731 |  |  |
| Total | 7 | 1.73678E6 |  |  |  |

At the 0.05 level, the population means are not significantly different

At the 0.05 level, the population means are not significantly different

At the 0.05 level, the population means are significantly different

Table S6-6 The significant difference between Cd treatments and Cd+Na treatments under warm temperature and high [CO_2_] conditions in rice roots.

|  | DF | Sum of Squares | Mean Square | F Value | Prob>F |
| --- | --- | --- | --- | --- | --- |
| (1) 0.2 mg Cd/L vs. 0.2 mg Cd Cd/L+0.2 g Na/L | | | | | |
| Model | 1 | 495.39169 | 495.39169 | 0.87201 | 0.38644 |
| Error | 6 | 3408.60794 | 568.10132 |  |  |
| Total | 7 | 3903.99963 |  |  |  |
| (2) 1.8 mg Cd/L vs. 1.8 mg Cd Cd/L+0.6 g Na/L | | | | | |
| Model | 1 | 495.39169 | 495.39169 | 0.87201 | 0.38644 |
| Error | 6 | 3408.60794 | 568.10132 |  |  |
| Total | 7 | 3903.99963 |  |  |  |
| (3) 5.4 mg Cd/L vs. 5.4 mg Cd Cd/L+1.1 g Na/L | | | | | |
| Model | 1 | 1.94241E6 | 1.94241E6 | 39.43651 | 7.58341E-4 |
| Error | 6 | 295525.14816 | 49254.19136 |  |  |
| Total | 7 | 2.23794E6 |  |  |  |

(1) At the 0.05 level, the population means are not significantly different

(2) At the 0.05 level, the population means are not significantly different

(3) At the 0.05 level, the population means are significantly different

Table S6-7 The significant difference between Cd treatments and Cd+Na treatments under cold temperature and ambient [CO_2_] conditions in rice shoots.

|  | DF | Sum of Squares | Mean Square | F Value | Prob>F |
| --- | --- | --- | --- | --- | --- |
| (1) 0.2 mg Cd/L vs. 0.2 mg Cd Cd/L+0.2 g Na/L | | | | | |
| Model | 1 | 2.76672 | 2.76672 | 3.32925 | 0.11786 |
| Error | 6 | 4.98622 | 0.83104 |  |  |
| Total | 7 | 7.75294 |  |  |  |
| (2) 1.8 mg Cd/L vs. 1.8 mg Cd Cd/L+0.6 g Na/L | | | | | |
| Model | 1 | 13.32113 | 13.32113 | 2.62528 | 0.1563 |
| Error | 6 | 30.44507 | 5.07418 |  |  |
| Total | 7 | 43.7662 |  |  |  |
| (3) 5.4 mg Cd/L vs. 5.4 mg Cd Cd/L+1.1 g Na/L | | | | | |
| Model | 1 | 323.3436 | 323.3436 | 44.86737 | 5.37279E-4 |
| Error | 6 | 43.23992 | 7.20665 |  |  |
| Total | 7 | 366.58352 |  |  |  |

(1) At the 0.05 level, the population means are not significantly different

(2) At the 0.05 level, the population means are not significantly different

(3) At the 0.05 level, the population means are significantly different

Table S6-8 The significant difference between Cd treatments and Cd+Na treatments under cold temperature and medium [CO_2_] conditions in rice shoots.

|  | DF | Sum of Squares | Mean Square | F Value | Prob>F |
| --- | --- | --- | --- | --- | --- |
| (1) 0.2 mg Cd/L vs. 0.2 mg Cd Cd/L+0.2 g Na/L | | | | | |
| Model | 1 | 2.15745 | 2.15745 | 0.48401 | 0.51265 |
| Error | 6 | 26.74502 | 4.4575 |  |  |
| Total | 7 | 28.90248 |  |  |  |
| (2) 1.8 mg Cd/L vs. 1.8 mg Cd Cd/L+0.6 g Na/L | | | | | |
| Model | 1 | 22.19982 | 22.19982 | 3.47677 | 0.11151 |
| Error | 6 | 38.31112 | 6.38519 |  |  |
| Total | 7 | 60.51094 |  |  |  |
| (3) 5.4 mg Cd/L vs. 5.4 mg Cd Cd/L+1.1 g Na/L | | | | | |
| Model | 1 | 25.80691 | 25.80691 | 1.3229 | 0.29384 |
| Error | 6 | 117.04727 | 19.50788 |  |  |
| Total | 7 | 142.85418 |  |  |  |

(1) At the 0.05 level, the population means are not significantly different

(2) At the 0.05 level, the population means are not significantly different

(3) At the 0.05 level, the population means are not significantly different

Table S6-9 The significant difference between Cd treatments and Cd+Na treatments under cold temperature and high [CO_2_] conditions in rice shoots.

|  | DF | Sum of Squares | Mean Square | F Value | Prob>F |
| --- | --- | --- | --- | --- | --- |
| (1) 0.2 mg Cd/L vs. 0.2 mg Cd Cd/L+0.2 g Na/L | | | | | |
| Model | 1 | 1.56436 | 1.56436 | 0.62813 | 0.45822 |
| Error | 6 | 14.94304 | 2.49051 |  |  |
| Total | 7 | 16.5074 |  |  |  |
| (2) 1.8 mg Cd/L vs. 1.8 mg Cd Cd/L+0.6 g Na/L | | | | | |
| Model | 1 | 0.21722 | 0.21722 | 0.03833 | 0.85125 |
| Error | 6 | 34.00387 | 5.66731 |  |  |
| Total | 7 | 34.22109 |  |  |  |
| (3) 5.4 mg Cd/L vs. 5.4 mg Cd Cd/L+1.1 g Na/L | | | | | |
| Model | 1 | 0.06148 | 0.06148 | 0.00206 | 0.96528 |
| Error | 6 | 179.17402 | 29.86234 |  |  |
| Total | 7 | 179.2355 |  |  |  |

(1) At the 0.05 level, the population means are not significantly different

(2) At the 0.05 level, the population means are not significantly different

(3) At the 0.05 level, the population means are not significantly different

Table S6-10 The significant difference between Cd treatments and Cd+Na treatments under warm temperature and ambient [CO_2_] conditions in rice shoots.

|  | DF | Sum of Squares | Mean Square | F Value | Prob>F |
| --- | --- | --- | --- | --- | --- |
| (1) 0.2 mg Cd/L vs. 0.2 mg Cd Cd/L+0.2 g Na/L | | | | | |
| Model | 1 | 5.50913 | 5.50913 | 1.38046 | 0.28453 |
| Error | 6 | 23.94475 | 3.99079 |  |  |
| Total | 7 | 29.45388 |  |  |  |
| (2) 1.8 mg Cd/L vs. 1.8 mg Cd Cd/L+0.6 g Na/L | | | | | |
| Model | 1 | 24.24228 | 24.24228 | 1.67134 | 0.24363 |
| Error | 6 | 87.0282 | 14.5047 |  |  |
| Total | 7 | 111.27047 |  |  |  |
| (3) 5.4 mg Cd/L vs. 5.4 mg Cd Cd/L+1.1 g Na/L | | | | | |
| Model | 1 | 2.99221 | 2.99221 | 0.57936 | 0.4754 |
| Error | 6 | 30.98794 | 5.16466 |  |  |
| Total | 7 | 33.98015 |  |  |  |

(1) At the 0.05 level, the population means are not significantly different

(2) At the 0.05 level, the population means are significantly different

(3) At the 0.05 level, the population means are not significantly different

Table S6-11 The significant difference between Cd treatments and Cd+Na treatments under warm temperature and medium [CO_2_] conditions in rice shoots.

|  | DF | Sum of Squares | Mean Square | F Value | Prob>F |
| --- | --- | --- | --- | --- | --- |
| (1) 0.2 mg Cd/L vs. 0.2 mg Cd Cd/L+0.2 g Na/L | | | | | |
| Model | 1 | 0.12039 | 0.12039 | 0.05003 | 0.83044 |
| Error | 6 | 14.43882 | 2.40647 |  |  |
| Total | 7 | 14.55921 |  |  |  |
| (2) 1.8 mg Cd/L vs. 1.8 mg Cd Cd/L+0.6 g Na/L | | | | | |
| Model | 1 | 9.50883 | 9.50883 | 3.27082 | 0.12052 |
| Error | 6 | 17.44303 | 2.90717 |  |  |
| Total | 7 | 26.95186 |  |  |  |
| (3) 5.4 mg Cd/L vs. 5.4 mg Cd Cd/L+1.1 g Na/L | | | | | |
| Model | 1 | 1.34953 | 1.34953 | 0.08023 | 0.7865 |
| Error | 6 | 100.92046 | 16.82008 |  |  |
| Total | 7 | 102.27 |  |  |  |

(1) At the 0.05 level, the population means are not significantly different

(2) At the 0.05 level, the population means are not significantly different

(3) At the 0.05 level, the population means are not significantly different

Table S6-12 The significant difference between Cd treatments and Cd+Na treatments under warm temperature and high [CO_2_] conditions in rice shoots.

|  | DF | Sum of Squares | Mean Square | F Value | Prob>F |
| --- | --- | --- | --- | --- | --- |
| (1) 0.2 mg Cd/L vs. 0.2 mg Cd Cd/L+0.2 g Na/L | | | | | |
| Model | 1 | 2.88532 | 2.88532 | 2.26536 | 0.183 |
| Error | 6 | 7.64201 | 1.27367 |  |  |
| Total | 7 | 10.52733 |  |  |  |
| (2) 1.8 mg Cd/L vs. 1.8 mg Cd Cd/L+0.6 g Na/L | | | | | |
| Model | 1 | 5.50565 | 5.50565 | 1.27546 | 0.30186 |
| Error | 6 | 25.89952 | 4.31659 |  |  |
| Total | 7 | 31.40517 |  |  |  |
| (3) 5.4 mg Cd/L vs. 5.4 mg Cd Cd/L+1.1 g Na/L | | | | | |
| Model | 1 | 14.72694 | 14.72694 | 6.60873 | 0.04229 |
| Error | 6 | 13.37043 | 2.22841 |  |  |
| Total | 7 | 28.09737 |  |  |  |

(1) At the 0.05 level, the population means are not significantly different

(2) At the 0.05 level, the population means are not significantly different

(3) At the 0.05 level, the population means are significantly different

**(3) The results of ANOVA and Tukey’s multiple range test were showed in Table S7&S8 and Figure 3, respectively.**

**Table S7** The significant difference between high/medium [CO_2_] and ambient [CO_2_] under same temperature.

Table 7-1 The significant difference between high/medium [CO_2_] and ambient [CO_2_] under cold temperature with 0.2 g/L Na treatment in rice roots.

|  | DF | Sum of Squares | Mean Square | F Value | Prob>F |
| --- | --- | --- | --- | --- | --- |
| Model | 2 | 3145003.81762 | 1572501.90881 | 7.24107 | 0.01336 |
| Error | 9 | 1954479.97719 | 217164.44191 |  |  |
| Total | 11 | 5099483.7948 |  |  |  |

At the 0.05 level, the population means are significantly different

Table 7-2 The significant difference between high/medium [CO_2_] and ambient [CO_2_] under cold temperature with 0.6 g/L Na treatment in rice roots.

|  | DF | Sum of Squares | Mean Square | F Value | Prob>F |
| --- | --- | --- | --- | --- | --- |
| Model | 2 | 1.43045E7 | 7152248.25215 | 12.42135 | 0.00258 |
| Error | 9 | 5182226.55412 | 575802.95046 |  |  |
| Total | 11 | 1.94867E7 |  |  |  |

At the 0.05 level, the population means are significantly different

Table 7-3 The significant difference between high/medium [CO_2_] and ambient [CO_2_] under cold temperature with 1.1 g/L Na treatment in rice roots.

|  | DF | Sum of Squares | Mean Square | F Value | Prob>F |
| --- | --- | --- | --- | --- | --- |
| Model | 2 | 1.30837E7 | 6541852.73616 | 1.07173 | 0.38238 |
| Error | 9 | 5.4936E7 | 6103994.6569 |  |  |
| Total | 11 | 6.80197E7 |  |  |  |

At the 0.05 level, the population means are not significantly different

Table 7-4 The significant difference between high/medium [CO_2_] and ambient [CO_2_] under warm temperature with 0.2 g/L Na treatment in rice roots.

|  | DF | Sum of Squares | Mean Square | F Value | Prob>F |
| --- | --- | --- | --- | --- | --- |
| Model | 2 | 3727693.90065 | 1863846.95033 | 7.2207 | 0.01346 |
| Error | 9 | 2323130.42727 | 258125.60303 |  |  |
| Total | 11 | 6050824.32792 |  |  |  |

At the 0.05 level, the population means are significantly different

Table 7-5 The significant difference between high/medium [CO_2_] and ambient [CO_2_] under warm temperature with 0.6 g/L Na treatment in rice roots.

|  | DF | Sum of Squares | Mean Square | F Value | Prob>F |
| --- | --- | --- | --- | --- | --- |
| Model | 2 | 1.79531E7 | 8976531.83477 | 29.00708 | 1.19218E-4 |
| Error | 9 | 2785140.1453 | 309460.01614 |  |  |
| Total | 11 | 2.07382E7 |  |  |  |

At the 0.05 level, the population means are significantly different

Table 7-6 The significant difference between high/medium [CO_2_] and ambient [CO_2_] under warm temperature with 1.1 g/L Na treatment in rice roots.

|  | DF | Sum of Squares | Mean Square | F Value | Prob>F |
| --- | --- | --- | --- | --- | --- |
| Model | 2 | 3.98311E7 | 1.99155E7 | 25.03661 | 2.10297E-4 |
| Error | 9 | 7159104.80414 | 795456.08935 |  |  |
| Total | 11 | 4.69902E7 |  |  |  |

At the 0.05 level, the population means are significantly different

Table 7-7 The significant difference between high/medium [CO_2_] and ambient [CO_2_] under cold temperature with 0.2 g/L Na treatment in rice shoots.

|  | DF | Sum of Squares | Mean Square | F Value | Prob>F |
| --- | --- | --- | --- | --- | --- |
| Model | 2 | 726485.45515 | 363242.72758 | 9.17966 | 0.00672 |
| Error | 9 | 356133.35817 | 39570.37313 |  |  |
| Total | 11 | 1082618.81332 |  |  |  |

At the 0.05 level, the population means are significantly different

Table 7-8 The significant difference between high/medium [CO_2_] and ambient [CO_2_] under cold temperature with 0.6 g/L Na treatment in rice shoots.

|  | DF | Sum of Squares | Mean Square | F Value | Prob>F |
| --- | --- | --- | --- | --- | --- |
| Model | 2 | 1149879.2825 | 574939.64125 | 7.99988 | 0.01008 |
| Error | 9 | 646817.06879 | 71868.5632 |  |  |
| Total | 11 | 1796696.3513 |  |  |  |

At the 0.05 level, the population means are significantly different

Table 7-9 The significant difference between high/medium [CO_2_] and ambient [CO_2_] under cold temperature with 1.1 g/L Na treatment in rice shoots.

|  | DF | Sum of Squares | Mean Square | F Value | Prob>F |
| --- | --- | --- | --- | --- | --- |
| Model | 2 | 9558147.06996 | 4779073.53498 | 8.10339 | 0.00971 |
| Error | 9 | 5307860.49841 | 589762.2776 |  |  |
| Total | 11 | 1.4866E7 |  |  |  |

At the 0.05 level, the population means are significantly different

Table 7-10 The significant difference between high/medium [CO_2_] and ambient [CO_2_] under warm temperature with 0.2 g/L Na treatment in rice shoots.

|  | DF | Sum of Squares | Mean Square | F Value | Prob>F |
| --- | --- | --- | --- | --- | --- |
| Model | 2 | 3571402.27592 | 1785701.13796 | 23.59039 | 2.636E-4 |
| Error | 9 | 681265.07685 | 75696.11965 |  |  |
| Total | 11 | 4252667.35277 |  |  |  |

At the 0.05 level, the population means are significantly different

Table 7-11 The significant difference between high/medium [CO_2_] and ambient [CO_2_] under warm temperature with 0.6 g/L Na treatment in rice shoots.

|  | DF | Sum of Squares | Mean Square | F Value | Prob>F |
| --- | --- | --- | --- | --- | --- |
| Model | 2 | 5103421.08149 | 2551710.54075 | 57.09605 | 7.6996E-6 |
| Error | 9 | 402223.86839 | 44691.54093 |  |  |
| Total | 11 | 5505644.94988 |  |  |  |

At the 0.05 level, the population means are significantly different

Table 7-12 The significant difference between high/medium [CO_2_] and ambient [CO_2_] under warm temperature with 1.1 g/L Na treatment in rice shoots.

|  | DF | Sum of Squares | Mean Square | F Value | Prob>F |
| --- | --- | --- | --- | --- | --- |
| Model | 2 | 3.48043E7 | 1.74022E7 | 16.91577 | 8.93623E-4 |
| Error | 9 | 9258781.56932 | 1028753.5077 |  |  |
| Total | 11 | 4.40631E7 |  |  |  |

At the 0.05 level, the population means are significantly different

Table 7-13 The significant difference between high/medium [CO_2_] and ambient [CO_2_] under cold temperature with 0.2 g Na/L+0.2 mg Cd/L treatment in rice roots.

|  | DF | Sum of Squares | Mean Square | F Value | Prob>F |
| --- | --- | --- | --- | --- | --- |
| Model | 2 | 2324446.46862 | 1162223.23431 | 3.04183 | 0.09791 |
| Error | 9 | 3438718.50366 | 382079.83374 |  |  |
| Total | 11 | 5763164.97228 |  |  |  |

At the 0.05 level, the population means are not significantly different

Table 7-14 The significant difference between high/medium [CO_2_] and ambient [CO_2_] under cold temperature with 0.6 g Na/L+1.8 mg Cd/L treatment in rice roots.

|  | DF | Sum of Squares | Mean Square | F Value | Prob>F |
| --- | --- | --- | --- | --- | --- |
| Model | 2 | 3120686.89603 | 1560343.44801 | 1.99514 | 0.19178 |
| Error | 9 | 7038643.43723 | 782071.49303 |  |  |
| Total | 11 | 1.01593E7 |  |  |  |

At the 0.05 level, the population means are not significantly different

Table 7-15 The significant difference between high/medium [CO_2_] and ambient [CO_2_] under cold temperature with 1.1 g Na/L+5.4 mg Cd/L treatment in rice roots.

|  | DF | Sum of Squares | Mean Square | F Value | Prob>F |
| --- | --- | --- | --- | --- | --- |
| Model | 2 | 2.56449E7 | 1.28225E7 | 14.91677 | 0.00139 |
| Error | 9 | 7736396.4271 | 859599.60301 |  |  |
| Total | 11 | 3.33813E7 |  |  |  |

At the 0.05 level, the population means are significantly different

Table 7-16 The significant difference between high/medium [CO_2_] and ambient [CO_2_] under warm temperature with 0.2 g Na/L+0.2 mg Cd/L treatment in rice roots.

|  | DF | Sum of Squares | Mean Square | F Value | Prob>F |
| --- | --- | --- | --- | --- | --- |
| Model | 2 | 1.57996E7 | 7899790.77867 | 23.30604 | 2.7595E-4 |
| Error | 9 | 3050631.1006 | 338959.01118 |  |  |
| Total | 11 | 1.88502E7 |  |  |  |

At the 0.05 level, the population means are significantly different

Table 7-17 The significant difference between high/medium [CO_2_] and ambient [CO_2_] under warm temperature with 0.6 g Na/L+1.8 mg Cd/L treatment in rice roots.

|  | DF | Sum of Squares | Mean Square | F Value | Prob>F |
| --- | --- | --- | --- | --- | --- |
| Model | 2 | 3.1752E7 | 1.5876E7 | 15.6126 | 0.00119 |
| Error | 9 | 9151847.8699 | 1016871.98554 |  |  |
| Total | 11 | 4.09039E7 |  |  |  |

At the 0.05 level, the population means are significantly different

Table 7-18 The significant difference between high/medium [CO_2_] and ambient [CO_2_] under warm temperature with 1.1 g Na/L+5.4 mg Cd/L treatment in rice roots.

|  | DF | Sum of Squares | Mean Square | F Value | Prob>F |
| --- | --- | --- | --- | --- | --- |
| Model | 2 | 7.30832E7 | 3.65416E7 | 12.81281 | 0.00233 |
| Error | 9 | 2.56676E7 | 2851957.97982 |  |  |
| Total | 11 | 9.87508E7 |  |  |  |

At the 0.05 level, the population means are significantly different

Table 7-19 The significant difference between high/medium [CO_2_] and ambient [CO_2_] under cold temperature with 0.2 g Na/L+0.2 mg Cd/L treatment in rice shoots.

|  | DF | Sum of Squares | Mean Square | F Value | Prob>F |
| --- | --- | --- | --- | --- | --- |
| Model | 2 | 1979018.80072 | 989509.40036 | 22.20963 | 3.30713E-4 |
| Error | 9 | 400978.57223 | 44553.17469 |  |  |
| Total | 11 | 2379997.37295 |  |  |  |

At the 0.05 level, the population means are significantly different

Table 7-20 The significant difference between high/medium [CO_2_] and ambient [CO_2_] under cold temperature with 0.6 g Na/L+1.8 mg Cd/L treatment in rice shoots.

|  | DF | Sum of Squares | Mean Square | F Value | Prob>F |
| --- | --- | --- | --- | --- | --- |
| Model | 2 | 2.30767E7 | 1.15384E7 | 26.80581 | 1.61862E-4 |
| Error | 9 | 3873985.3238 | 430442.81376 |  |  |
| Total | 11 | 2.69507E7 |  |  |  |

At the 0.05 level, the population means are significantly different

Table 7-21 The significant difference between high/medium [CO_2_] and ambient [CO_2_] under cold temperature with 1.1 g Na/L+5.4 mg Cd/L treatment in rice shoots.

|  | DF | Sum of Squares | Mean Square | F Value | Prob>F |
| --- | --- | --- | --- | --- | --- |
| Model | 2 | 8.03352E7 | 4.01676E7 | 87.897 | 1.24164E-6 |
| Error | 9 | 4112863.7532 | 456984.86147 |  |  |
| Total | 11 | 8.44481E7 |  |  |  |

At the 0.05 level, the population means are significantly different

Table 7-22 The significant difference between high/medium [CO_2_] and ambient [CO_2_] under warm temperature with 0.2 g Na/L+0.2 mg Cd/L treatment in rice shoots.

|  | DF | Sum of Squares | Mean Square | F Value | Prob>F |
| --- | --- | --- | --- | --- | --- |
| Model | 2 | 413482.18453 | 206741.09227 | 6.52782 | 0.01771 |
| Error | 9 | 285037.14987 | 31670.79443 |  |  |
| Total | 11 | 698519.3344 |  |  |  |

At the 0.05 level, the population means are significantly different

Table 7-23 The significant difference between high/medium [CO_2_] and ambient [CO_2_] under warm temperature with 0.6 g Na/L+1.8 mg Cd/L treatment in rice shoots.

|  | DF | Sum of Squares | Mean Square | F Value | Prob>F |
| --- | --- | --- | --- | --- | --- |
| Model | 2 | 5606378.74037 | 2803189.37019 | 5.58749 | 0.02645 |
| Error | 9 | 4515216.14078 | 501690.68231 |  |  |
| Total | 11 | 1.01216E7 |  |  |  |

At the 0.05 level, the population means are significantly different

Table 7-24 The significant difference between high/medium [CO_2_] and ambient [CO_2_] under warm temperature with 1.1 g Na/L+5.4 mg Cd/L treatment in rice shoots.

|  | DF | Sum of Squares | Mean Square | F Value | Prob>F |
| --- | --- | --- | --- | --- | --- |
| Model | 2 | 2.12894E7 | 1.06447E7 | 13.58333 | 0.00191 |
| Error | 9 | 7052936.28789 | 783659.58754 |  |  |
| Total | 11 | 2.83423E7 |  |  |  |

At the 0.05 level, the population means are significantly different

**Table S8** The significant difference between Na treatments and Cd+Na treatments under same temperature and [CO_2_] conditions.

Table 8-1 The significant difference between Na treatments and Cd+Na treatments under cold temperature and ambient [CO_2_] conditions in rice roots.

|  | DF | Sum of Squares | Mean Square | F Value | Prob>F |
| --- | --- | --- | --- | --- | --- |
| (1) 0.2 g Na/L vs. 0.2 mg Cd Cd/L+0.2 g Na/L | | | | | |
| Model | 1 | 9.71645E6 | 9.71645E6 | 41.38636 | 6.67004E-4 |
| Error | 6 | 1.40865E6 | 234774.17377 |  |  |
| Total | 7 | 1.11251E7 |  |  |  |
| (2) 0.6 g Na/L vs. 1.8 mg Cd Cd/L+0.6 g Na/L | | | | | |
| Model | 1 | 9.76344E6 | 9.76344E6 | 13.48066 | 0.01043 |
| Error | 6 | 4.34553E6 | 724255.79339 |  |  |
| Total | 7 | 1.4109E7 |  |  |  |
| (3) 1.1 g Na/L vs. 5.4 mg Cd Cd/L+1.1 g Na/L | | | | | |
| Model | 1 | 1.87316E7 | 1.87316E7 | 2.68479 | 0.15242 |
| Error | 6 | 4.18616E7 | 6.97693E6 |  |  |
| Total | 7 | 6.05931E7 |  |  |  |

(1) At the 0.05 level, the population means are significantly different

(2) At the 0.05 level, the population means are significantly different

(3) At the 0.05 level, the population means are not significantly different

Table 8-2 The significant difference between Na treatments and Cd+Na treatments under cold temperature and medium [CO_2_] conditions in rice roots.

|  | DF | Sum of Squares | Mean Square | F Value | Prob>F |
| --- | --- | --- | --- | --- | --- |
| (1) 0.2 g Na/L vs. 0.2 mg Cd Cd/L+0.2 g Na/L | | | | | |
| Model | 1 | 1.17928E7 | 1.17928E7 | 49.77943 | 4.05723E-4 |
| Error | 6 | 1.4214E6 | 236900.32884 |  |  |
| Total | 7 | 1.32142E7 |  |  |  |
| (2) 0.6 g Na/L vs. 1.8 mg Cd Cd/L+0.6 g Na/L | | | | | |
| Model | 1 | 2.1189E7 | 2.1189E7 | 25.43603 | 0.00235 |
| Error | 6 | 4.99818E6 | 833029.53779 |  |  |
| Total | 7 | 2.61871E7 |  |  |  |
| (3) 1.1 g Na/L vs. 5.4 mg Cd Cd/L+1.1 g Na/L | | | | | |
| Model | 1 | 2.2612E6 | 2.2612E6 | 1.11564 | 0.33152 |
| Error | 6 | 1.21609E7 | 2.02681E6 |  |  |
| Total | 7 | 1.44221E7 |  |  |  |

(1) At the 0.05 level, the population means are significantly different

(2) At the 0.05 level, the population means are significantly different

(3) At the 0.05 level, the population means are not significantly different

Table 8-3 The significant difference between Na treatments and Cd+Na treatments under cold temperature and high [CO_2_] conditions in rice roots.

|  | DF | Sum of Squares | Mean Square | F Value | Prob>F |
| --- | --- | --- | --- | --- | --- |
| (1) 0.2 g Na/L vs. 0.2 mg Cd Cd/L+0.2 g Na/L | | | | | |
| Model | 1 | 8.78939E6 | 8.78939E6 | 20.5748 | 0.00395 |
| Error | 6 | 2.56315E6 | 427191.91086 |  |  |
| Total | 7 | 1.13525E7 |  |  |  |
| (2) 0.6 g Na/L vs. 1.8 mg Cd Cd/L+0.6 g Na/L | | | | | |
| Model | 1 | 2.56282E7 | 2.56282E7 | 53.44477 | 3.34294E-4 |
| Error | 6 | 2.87716E6 | 479526.33405 |  |  |
| Total | 7 | 2.85053E7 |  |  |  |
| (3) 1.1 g Na/L vs. 5.4 mg Cd Cd/L+1.1 g Na/L | | | | | |
| Model | 1 | 8.40042E6 | 8.40042E6 | 5.82693 | 0.0523 |
| Error | 6 | 8.64993E6 | 1.44166E6 |  |  |
| Total | 7 | 1.70504E7 |  |  |  |

(1) At the 0.05 level, the population means are significantly different

(2) At the 0.05 level, the population means are significantly different

(3) At the 0.05 level, the population means are not significantly different

Table 8-4 The significant difference between Na treatments and Cd+Na treatments under warm temperature and ambient [CO_2_] conditions in rice roots.

|  | DF | Sum of Squares | Mean Square | F Value | Prob>F |
| --- | --- | --- | --- | --- | --- |
| (1) 0.2 g Na/L vs. 0.2 mg Cd Cd/L+0.2 g Na/L | | | | | |
| Model | 1 | 3.00404E6 | 3.00404E6 | 34.15454 | 0.00111 |
| Error | 6 | 527725.21589 | 87954.20265 |  |  |
| Total | 7 | 3.53176E6 |  |  |  |
| (2) 0.6 g Na/L vs. 1.8 mg Cd Cd/L+0.6 g Na/L | | | | | |
| Model | 1 | 6.17865E6 | 6.17865E6 | 28.2067 | 0.00181 |
| Error | 6 | 1.3143E6 | 219049.19245 |  |  |
| Total | 7 | 7.49295E6 |  |  |  |
| (3) 1.1 g Na/L vs. 5.4 mg Cd Cd/L+1.1 g Na/L | | | | | |
| Model | 1 | 2.19446E6 | 2.19446E6 | 0.87507 | 0.38566 |
| Error | 6 | 1.50465E7 | 2.50775E6 |  |  |
| Total | 7 | 1.7241E7 |  |  |  |

(1) At the 0.05 level, the population means are significantly different

(2) At the 0.05 level, the population means are significantly different

(3) At the 0.05 level, the population means are not significantly different

Table 8-5 The significant difference between Na treatments and Cd+Na treatments under warm temperature and medium [CO_2_] conditions in rice roots.

|  | DF | Sum of Squares | Mean Square | F Value | Prob>F |
| --- | --- | --- | --- | --- | --- |
| (1) 0.2 g Na/L vs. 0.2 mg Cd Cd/L+0.2 g Na/L | | | | | |
| Model | 1 | 1.07474E7 | 1.07474E7 | 16.20326 | 0.00692 |
| Error | 6 | 3.97972E6 | 663286.33652 |  |  |
| Total | 7 | 1.47271E7 |  |  |  |
| (2) 0.6 g Na/L vs. 1.8 mg Cd Cd/L+0.6 g Na/L | | | | | |
| Model | 1 | 1.97388E7 | 1.97388E7 | 33.67319 | 0.00115 |
| Error | 6 | 3.51712E6 | 586186.24169 |  |  |
| Total | 7 | 2.32559E7 |  |  |  |
| (3) 1.1 g Na/L vs. 5.4 mg Cd Cd/L+1.1 g Na/L | | | | | |
| Model | 1 | 2.59156E7 | 2.59156E7 | 21.19399 | 0.00368 |
| Error | 6 | 7.33668E6 | 1.22278E6 |  |  |
| Total | 7 | 3.32523E7 |  |  |  |

(1) At the 0.05 level, the population means are significantly different

(2) At the 0.05 level, the population means are significantly different

(3) At the 0.05 level, the population means are significantly different

Table 8-6 The significant difference between Na treatments and Cd+Na treatments under warm temperature and high [CO_2_] conditions in rice roots.

|  | DF | Sum of Squares | Mean Square | F Value | Prob>F |
| --- | --- | --- | --- | --- | --- |
| (1) 0.2 g Na/L vs. 0.2 mg Cd Cd/L+0.2 g Na/L | | | | | |
| Model | 1 | 411949.05947 | 411949.05947 | 2.8531 | 0.14216 |
| Error | 6 | 866318.29284 | 144386.38214 |  |  |
| Total | 7 | 1.27827E6 |  |  |  |
| (2) 0.6 g Na/L vs. 1.8 mg Cd Cd/L+0.6 g Na/L | | | | | |
| Model | 1 | 2.97239E6 | 2.97239E6 | 2.50991 | 0.16422 |
| Error | 6 | 7.10558E6 | 1.18426E6 |  |  |
| Total | 7 | 1.0078E7 |  |  |  |
| (3) 1.1 g Na/L vs. 5.4 mg Cd Cd/L+1.1 g Na/L | | | | | |
| Model | 1 | 652129.41842 | 652129.41842 | 0.37466 | 0.56294 |
| Error | 6 | 1.04435E7 | 1.74059E6 |  |  |
| Total | 7 | 1.10956E7 |  |  |  |

(1) At the 0.05 level, the population means are not significantly different

(2) At the 0.05 level, the population means are not significantly different

(3) At the 0.05 level, the population means are not significantly different

Table 8-7 The significant difference between Na treatments and Cd+Na treatments under cold temperature and ambient [CO_2_] conditions in rice shoots.

|  | DF | Sum of Squares | Mean Square | F Value | Prob>F |
| --- | --- | --- | --- | --- | --- |
| (1) 0.2 g Na/L vs. 0.2 mg Cd Cd/L+0.2 g Na/L | | | | | |
| Model | 1 | 2.43265E6 | 2.43265E6 | 220.00143 | 5.90666E-6 |
| Error | 6 | 66344.66519 | 11057.4442 |  |  |
| Total | 7 | 2.499E6 |  |  |  |
| (2) 0.6 g Na/L vs. 1.8 mg Cd Cd/L+0.6 g Na/L | | | | | |
| Model | 1 | 2.70465E7 | 2.70465E7 | 123.61809 | 3.15496E-5 |
| Error | 6 | 1.31274E6 | 218790.51014 |  |  |
| Total | 7 | 2.83592E7 |  |  |  |
| (3) 1.1 g Na/L vs. 5.4 mg Cd Cd/L+1.1 g Na/L | | | | | |
| Model | 1 | 5.3589E7 | 5.3589E7 | 81.58349 | 1.03163E-4 |
| Error | 6 | 3.94116E6 | 656860.67196 |  |  |
| Total | 7 | 5.75301E7 |  |  |  |

At the 0.05 level, the population means are significantly different

At the 0.05 level, the population means are significantly different

At the 0.05 level, the population means are significantly different

Table 8-8 The significant difference between Na treatments and Cd+Na treatments under cold temperature and medium [CO_2_] conditions in rice shoots.

|  | DF | Sum of Squares | Mean Square | F Value | Prob>F |
| --- | --- | --- | --- | --- | --- |
| (1) 0.2 g Na/L vs. 0.2 mg Cd Cd/L+0.2 g Na/L | | | | | |
| Model | 1 | 750947.1523 | 750947.1523 | 8.50289 | 0.02677 |
| Error | 6 | 529900.45774 | 88316.74296 |  |  |
| Total | 7 | 1.28085E6 |  |  |  |
| (2) 0.6 g Na/L vs. 1.8 mg Cd Cd/L+0.6 g Na/L | | | | | |
| Model | 1 | 3.39157E6 | 3.39157E6 | 14.81891 | 0.00846 |
| Error | 6 | 1.37321E6 | 228868.02288 |  |  |
| Total | 7 | 4.76478E6 |  |  |  |
| (3) 1.1 g Na/L vs. 5.4 mg Cd Cd/L+1.1 g Na/L | | | | | |
| Model | 1 | 1.53359E7 | 1.53359E7 | 38.05833 | 8.33206E-4 |
| Error | 6 | 2.41775E6 | 402957.79982 |  |  |
| Total | 7 | 1.77536E7 |  |  |  |

(1) At the 0.05 level, the population means are significantly different

(2) At the 0.05 level, the population means are significantly different

(3) At the 0.05 level, the population means are significantly different

Table 8-9 The significant difference between Na treatments and Cd+Na treatments under cold temperature and high [CO_2_] conditions in rice shoots.

|  | DF | Sum of Squares | Mean Square | F Value | Prob>F |
| --- | --- | --- | --- | --- | --- |
| (1) 0.2 g Na/L vs. 0.2 mg Cd Cd/L+0.2 g Na/L | | | | | |
| Model | 1 | 926020.22112 | 926020.22112 | 34.53864 | 0.00108 |
| Error | 6 | 160866.80747 | 26811.13458 |  |  |
| Total | 7 | 1.08689E6 |  |  |  |
| (2) 0.6 g Na/L vs. 1.8 mg Cd Cd/L+0.6 g Na/L | | | | | |
| Model | 1 | 3.81027E6 | 3.81027E6 | 12.45965 | 0.01237 |
| Error | 6 | 1.83485E6 | 305808.53241 |  |  |
| Total | 7 | 5.64512E6 |  |  |  |
| (3) 1.1 g Na/L vs. 5.4 mg Cd Cd/L+1.1 g Na/L | | | | | |
| Model | 1 | 1.6118E6 | 1.6118E6 | 3.15852 | 0.12586 |
| Error | 6 | 3.06181E6 | 510302.23681 |  |  |
| Total | 7 | 4.67361E6 |  |  |  |

(1) At the 0.05 level, the population means are significantly different

(2) At the 0.05 level, the population means are significantly different

(3) At the 0.05 level, the population means are not significantly different

Table 8-10 The significant difference between Na treatments and Cd+Na treatments under warm temperature and ambient [CO_2_] conditions in rice shoots.

|  | DF | Sum of Squares | Mean Square | F Value | Prob>F |
| --- | --- | --- | --- | --- | --- |
| (1) 0.2 g Na/L vs. 0.2 mg Cd Cd/L+0.2 g Na/L | | | | | |
| Model | 1 | 901367.77265 | 901367.77265 | 21.35606 | 0.00361 |
| Error | 6 | 253239.95201 | 42206.65867 |  |  |
| Total | 7 | 1.15461E6 |  |  |  |
| (2) 0.6 g Na/L vs. 1.8 mg Cd Cd/L+0.6 g Na/L | | | | | |
| Model | 1 | 1.03876E7 | 1.03876E7 | 42.48264 | 6.22027E-4 |
| Error | 6 | 1.46708E6 | 244513.59971 |  |  |
| Total | 7 | 1.18547E7 |  |  |  |
| (3) 1.1 g Na/L vs. 5.4 mg Cd Cd/L+1.1 g Na/L | | | | | |
| Model | 1 | 4.78265E6 | 4.78265E6 | 3.38796 | 0.11528 |
| Error | 6 | 8.46998E6 | 1.41166E6 |  |  |
| Total | 7 | 1.32526E7 |  |  |  |

(1) At the 0.05 level, the population means are significantly different

(2) At the 0.05 level, the population means are significantly different

(3) At the 0.05 level, the population means are not significantly different

Table 8-11 The significant difference between Na treatments and Cd+Na treatments under warm temperature and medium [CO_2_] conditions in rice shoots.

|  | DF | Sum of Squares | Mean Square | F Value | Prob>F |
| --- | --- | --- | --- | --- | --- |
| (1) 0.2 g Na/L vs. 0.2 mg Cd Cd/L+0.2 g Na/L | | | | | |
| Model | 1 | 1.41018E6 | 1.41018E6 | 22.41155 | 0.00321 |
| Error | 6 | 377531.27648 | 62921.87941 |  |  |
| Total | 7 | 1.78771E6 |  |  |  |
| (2) 0.6 g Na/L vs. 1.8 mg Cd Cd/L+0.6 g Na/L | | | | | |
| Model | 1 | 1.44725E7 | 1.44725E7 | 29.3036 | 0.00164 |
| Error | 6 | 2.96329E6 | 493881.111 |  |  |
| Total | 7 | 1.74358E7 |  |  |  |
| (3) 1.1 g Na/L vs. 5.4 mg Cd Cd/L+1.1 g Na/L | | | | | |
| Model | 1 | 1.33721E7 | 1.33721E7 | 15.03566 | 0.00819 |
| Error | 6 | 5.33617E6 | 889360.87755 |  |  |
| Total | 7 | 1.87083E7 |  |  |  |

(1) At the 0.05 level, the population means are significantly different

(2) At the 0.05 level, the population means are significantly different

(3) At the 0.05 level, the population means are significantly different

Table 8-12 The significant difference between Na treatments and Cd+Na treatments under warm temperature and high [CO_2_] conditions in rice shoots.

|  | DF | Sum of Squares | Mean Square | F Value | Prob>F |
| --- | --- | --- | --- | --- | --- |
| (1) 0.2 g Na/L vs. 0.2 mg Cd Cd/L+0.2 g Na/L | | | | | |
| Model | 1 | 1.10979E6 | 1.10979E6 | 19.84543 | 0.00431 |
| Error | 6 | 335530.99823 | 55921.83304 |  |  |
| Total | 7 | 1.44532E6 |  |  |  |
| (2) 0.6 g Na/L vs. 1.8 mg Cd Cd/L+0.6 g Na/L | | | | | |
| Model | 1 | 7.86659E6 | 7.86659E6 | 96.90471 | 6.33476E-5 |
| Error | 6 | 487071.74491 | 81178.62415 |  |  |
| Total | 7 | 8.35366E6 |  |  |  |
| (3) 1.1 g Na/L vs. 5.4 mg Cd Cd/L+1.1 g Na/L | | | | | |
| Model | 1 | 1.06745E7 | 1.06745E7 | 25.56169 | 0.00232 |
| Error | 6 | 2.50557E6 | 417595.76028 |  |  |
| Total | 7 | 1.318E7 |  |  |  |

(1) At the 0.05 level, the population means are significantly different

(2) At the 0.05 level, the population means are significantly different

(3) At the 0.05 level, the population means are significantly different
